# P2X7 receptor induces mitochondrial failure in monocytes and compromises NLRP3 inflammasome activation during sepsis

**DOI:** 10.1038/s41467-019-10626-x

**Published:** 2019-06-20

**Authors:** Juan José Martínez-García, Helios Martínez-Banaclocha, Diego Angosto-Bazarra, Carlos de Torre-Minguela, Alberto Baroja-Mazo, Cristina Alarcón-Vila, Laura Martínez-Alarcón, Joaquín Amores-Iniesta, Fátima Martín-Sánchez, Giovanni A. Ercole, Carlos M. Martínez, Ada González-Lisorge, José Fernández-Pacheco, Piedad Martínez-Gil, Sahil Adriouch, Friedrich Koch-Nolte, Juan Luján, Francisco Acosta-Villegas, Pascual Parrilla, Carlos García-Palenciano, Pablo Pelegrin

**Affiliations:** 1 Unidad de Inflamación Molecular y Cirugía Experimental, Instituto Murciano de Investigación Biosanitaria IMIB-Arrixaca, Hospital Clínico Universitario Virgen de la Arrixaca, Murcia, 30120 Spain; 2 Unidad de Reanimación, Hospital Clínico Universitario Virgen de la Arrixaca, Murcia, 30120 Spain; 3 Plataforma de Patología, Instituto Murciano de Investigación Biosanitaria IMIB-Arrixaca, Murcia, 30120 Spain; 4 Normandie University, UNIROUEN, INSERM, U1234, Rouen, 76183 France; 5 Institute of Immunology, University Medical Center Hamburg-Eppendorf, Hamburg, D-20246 Germany; 6 Servicio de Cirugía General, Hospital Clínico Universitario Virgen de la Arrixaca, Murcia, 30120 Spain

**Keywords:** Inflammasome, Bacterial infection

## Abstract

Sepsis is characterized by a systemic inflammatory response followed by immunosuppression of the host. Metabolic defects and mitochondrial failure are common in immunocompromised patients with sepsis. The NLRP3 inflammasome is important for establishing an inflammatory response after activation by the purinergic P2X7 receptor. Here, we study a cohort of individuals with intra-abdominal origin sepsis and show that patient monocytes have impaired NLRP3 activation by the P2X7 receptor. Furthermore, most sepsis-related deaths are among patients whose NLRP3 activation is profoundly altered. In monocytes from sepsis patients, the P2X7 receptor is associated with mitochondrial dysfunction. Furthermore, activation of the P2X7 receptor results in mitochondrial damage, which in turn inhibits NLRP3 activation by HIF-1α. We show that mortality increases in a mouse model of sepsis when the P2X7 receptor is activated in vivo. These data reveal a molecular mechanism initiated by the P2X7 receptor that contributes to NLRP3 impairment during infection.

## Abstract (web summary)

Systemic sepsis is a potentially life-threatening illness and immunocompromised individuals are especially vulnerable. Here, using a cohort of patients with intra-abdominal origin sepsis, the authors show an important role for the NLRP3 inflammasome in establishing a host response, and NLRP3 dysfunction is a common feature of sepsis mortality.

## Introduction

The response of the immune system during sepsis is a complex dynamic process involving an initial inflammatory response followed by immune deactivation. Exacerbation of any of these responses compromises the life of sepsis patients. The initial production of proinflammatory cytokines aims to protect the host from invading pathogens, but it can also damage non-infected tissues and lead to the dysfunction of different organs and systems^1^. Immunosuppression after the inflammatory response is a compensatory response aimed at protecting the host from the excess production of cytokines and other inflammatory factors; however, in sepsis there is a profound leukocyte deactivation that is related to complications in critically ill patients, and which leads to secondary fatal infections and the majority of deaths associated with sepsis^2^. Changes in the metabolism of leukocytes during sepsis are associated with the different immune responses in sepsis, whereby the glycolytic pathway is upregulated during the inflammatory response, while defects in both glycolysis and mitochondrial respiration lead to immunosuppression^3,4^. However, the molecular mechanisms in the host that initiate and regulate immunoparalysis in human sepsis are still poorly understood, although epigenetic modifications and interleukin (IL)-33 may contribute to long-term immunosuppression^5,6^.

Pattern recognition receptors in innate immune cells are able to recognize microbial-associated or damage-associated molecules and initiate a proinflammatory response^7^. Inflammasomes are important signaling complexes formed by a subgroup of intracellular pattern recognition receptors that activate caspase-1. Caspase-1 promotes a specific type of cell death called pyroptosis, and the release of the proinflammatory cytokines IL-1β, IL-18, and the alarmin high mobility group protein B1 (HMGB1), which are all involved in the inflammatory response of sepsis^8–11^. A promiscuous type of inflammasome is formed by the nucleotide-binding domain, leucine-rich repeat, and pyrin domain-containing protein 3 (NLRP3) that can be activated in response to different microbial-associated or damage-associated molecules, as elevated extracellular concentrations of the nucleotide adenosine triphosphate (ATP) signaling through the P2X purinoceptor 7 (P2X7)^12,13^. Upon activation, NLRP3 oligomerizes and recruits the apoptotic speck-like protein with a caspase-activating domain (ASC) into large oligomers, that can be found in biological fluids upon inflammasome activation and pyroptosis execution^14,15^.

The dynamic of the inflammatory response from proinflammatory state to immunosuppressive state during the development of sepsis is also reflected in the inflammasome. The NLRP3 inflammasome components are overexpressed in human leukocytes from septic patients^16^, and a defective function of the NLRP3 inflammasome has also been found as an immunological feature in septic patients^17^. Animal models of sepsis reflects the initial inflammatory response and during this phase a deficiency in NLRP3 augments survival^18,19^. However, the exact effects of sepsis when induced in P2X7-receptor-deficient mice are still the cause of some debate, because different studies have found that it can result in either higher mortality or survival^20–22^, thus denoting the significant variability in mortality in mouse models^23^. Therefore, further research is needed into how the P2X7 receptor controls NLRP3 inflammasome in sepsis, and whether these proinflammatory pathways can participate in the immunosuppression that occurs in human sepsis. In the present study, we assess the activation of NLRP3 inflammasome by the P2X7 receptor in blood leukocytes from a cohort of clinically relevant intra-abdominal origin sepsis patients and then carry out a follow-up analysis of the same individuals after sepsis resolution. Our results show that ATP-induced NLRP3 inflammasome does not activate correctly in the blood leukocytes of sepsis patients. We also identify P2X7 receptor as a modulator of mitochondrial depolarization in monocytes, which in turn leads to NLRP3 immunoparalysis.

## Results

### Inflammasome markers are elevated in sepsis

**Fig. 1:**
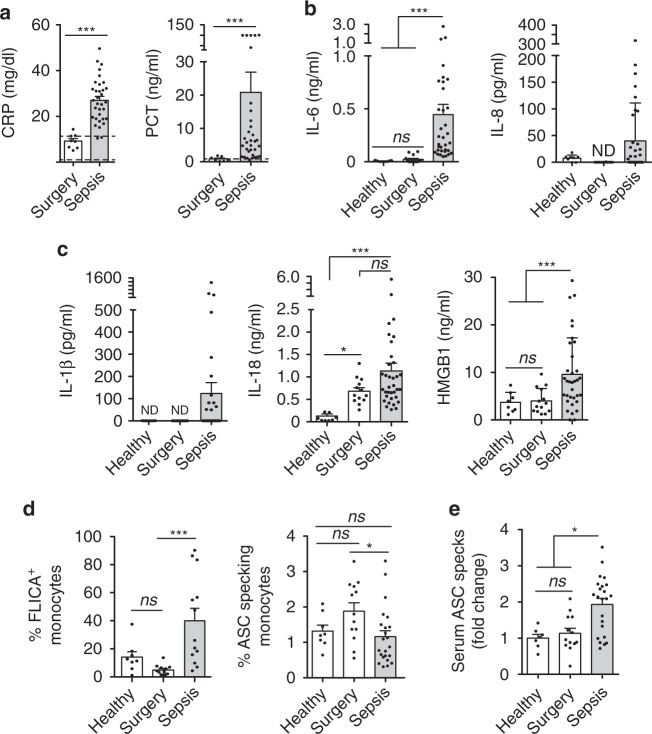
Sepsis patients present with elevated inflammasome markers. **a**–**c** Plasma concentration of CRP, PCT **a**, IL-6, IL-8 **b**, IL-1β, IL-18, and HMGB1 **c** in healthy controls, abdominal surgery patients within the first 24 h after surgery and intra-abdominal origin septic patients within the first 24 h of admission to the surgical critical unit; dotted lines in **a** represent standard threshold for CRP and PCT in healthy population. **d** Percentage of monocytes from healthy controls and septic patient positives for active caspase-1 labeled with FLICA (left) or positives for intracellular ASC specks (right). **e** Fold increase of circulating ASC specks in the plasma of surgery controls and septic patients compared with healthy controls. Each dot represents an individual patient; average ± standard error is represented in all panels; exact *n* number for each panel is presented in Source Data file; *p < 0.05; **p < 0.01; ***p < 0.001; *ns*, no significant difference (*p* > 0.05); Mann–Whitney test for **a**; Kruskal–Wallis test for **b**–**e**

We analyzed a cohort of intra-abdominal origin septic patients (*n* = 35, Supplementary Table 1), who presented elevated levels of C-reactive protein (CRP) and procalcitonin (PCT) in their plasma 24 h after sepsis initiation, when compared to a control group of abdominal surgery patients that had not developed sepsis (Fig. 1a). Septic patients presented an average acute physiology and chronic health evaluation (APACHE II) of 18.6 ± 7.7 (range 5–52, *n* = 35, average ± standard error), and a sequential organ failure assessment (SOFA) of 6.4 ± 2.8 (range 2–12, *n* = 35), with dysfunction of one or more vital organs together with an increase in neutrophils, lactate, NT-proBNP, creatinine, bilirubin, and fibrinogen (Supplementary Fig. 1 and Supplementary Tables 1 and 2). In contrast, the amounts of hemoglobin and bicarbonate were lower than the standards found in healthy individuals (Supplementary Fig. 1). As expected, IL-6 and IL-8 were also higher in the plasma of septic patients when compared to control surgery and healthy groups (Fig. 1b). However, no IL-12, IL-4, IFNγ, IL-2, and TNF-α were found in the plasma of septic patients with the exception of three septic individuals who presented measurable concentrations of TNF-α (Supplementary Table 3). On the other hand, the inflammasome-related cytokines IL-1β, IL-18, and the alarmin HMGB1 were higher in septic patients than in the control groups (Fig. 1c). Elevated concentration of IL-18 in plasma has been associated with mortality in sepsis^24^, however, the concentration of IL-18 in our septic cohort was not associated with death (1.24 ± 0.25 vs. 1.02 ± 0.17 ng/ml survival *n* = 23 vs. non-survival *n* = 12 septic patients, respectively, *p* = 0.7191 Mann–Whitney test, with an area under the curve in the receiver operating characteristic (ROC) analysis of 0.539 ± 0.098). We were also unable to find any correlation between cytokine concentration in the plasma and sepsis mortality. The percentage of monocytes with active caspase-1 was found to be higher in septic patients than in healthy controls, despite the fact that monocytes from septic patients did not present an increase in intracellular ASC speck formation (Fig. 1d), which coincides with evidence that activation of the NLRP3 inflammasome in human monocytes by the alternative pathway does not result in ASC specking^25^. However, circulating ASC specks in plasma were higher in septic patients than in control surgery and healthy groups (Fig. 1e), thus confirming our previous results showing that ASC specks in the plasma increase during systemic inflammation^14^. During sepsis ASC specks may be released from pyroptotic monocytes, other activated leukocytes, endothelial cells, or cardiomyocytes, that have all shown to release IL-1β in sepsis^26,27^. Altogether these data show that during the initial phase of sepsis there is an activation of the inflammasome that contributes to the cytokine storm, thus confirming previous studies, which show that septic patients present an upregulation of inflammasome genes in peripheral blood mononuclear cells (PBMCs)^16,28^ and elevated IL-18 in blood^24,29^.

### Sepsis compromises inflammasome activation

**Fig. 2:**
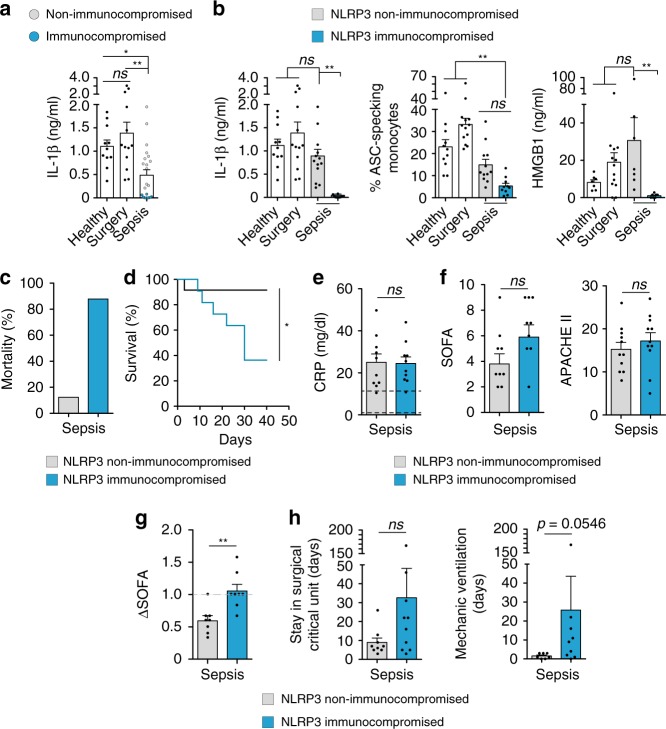
Inflammasome activation is compromised in septic patients with high mortality. **a** ELISA for IL-1β in PBMC supernatants and percentage of monocytes with intracellular ASC specks from septic patients within the first 24 h of admission to the surgical critical unit and control groups after NLRP3 inflammasome activation by LPS (1 μg/ml, 2 h) and ATP (3 mM, 30 min) treatment. There is a group of septic patients that release low or no IL-1β (blue dots). **b** ELISA for IL-1β and HMGB1 in PBMC supernatants and percentage of monocytes with intracellular ASC specks from control groups and septic patients treated as in **a**, separating septic patients into two groups: NLRP3 non-immunocompromised (gray bar) and immunocompromised (blue bar). **c** Percentage of mortality in NLRP3 non-immunocompromised (gray bar) and immunocompromised (blue bar) septic patients with regard to the total mortality of septic patients. **d** Kaplan–Meier representation of NLRP3 non-immunocompromised (gray line) and immunocompromised (blue line) septic patients’ survival. **e** Concentration of C-reactive protein (CRP) in plasma of NLRP3 non-immunocompromised (gray bar) and immunocompromised (blue bar) septic patients at day 1; dotted lines represent threshold concentration of CRP for healthy population. **f** SOFA and APACHEII indexes in NLRP3 non-immunocompromised (gray bar) and immunocompromised (blue bar) septic patients at day 1. **g** ΔSOFA calculated as the variation in SOFA between day 5 and day 1, gray dotted line represents no change with regard to day 1. **h** Number of days admitted to the Surgical Critical Unit and number of days of mechanical ventilation for NLRP3 non-immunocompromised (gray bar) and immunocompromised (blue bar) septic patients. Each dot represents an individual patient; average ± standard error is represented in panels **a**, **b**, **e**–**h**; exact *n* number for each panel is presented in Source Data file; *p < 0.05; **p < 0.01; ***p < 0.001; *ns*, no significant difference (*p* > 0.05); Kruskal–Wallis test for **a** and **b**; Log-rank test for **d**; and Mann–Whitney test for **e**–**h**

Despite the elevated concentration of IL-1β found in the plasma of septic patients, PBMCs from septic patients presented a decreased release of IL-1β, when LPS and ATP stimulation activated NLRP3 inflammasome (Fig. 2a), which corroborates a previous study where NLRP3 activation by monosodium urate crystals was inhibited in PBMCs from septic patients^17^. However, we found that the release of IL-1β was non-detectable or very low in some septic patients, who also presented a decreased release of HMGB1 and formation of ASC specks in monocytes after NLRP3 stimulation (Fig. 2b, Supplementary Fig. 2a, b). These patients also had deficient production of IL-6, TNF-α, and IL-8 (Supplementary Fig. 2c, d). The defect in NLRP3 activation in a subset of septic patients was not due to differences in inflammasome gene expression (Supplementary Fig. 2e). A ROC analysis showed that the release of IL-1β and the formation of ASC specks differentiated septic patients from healthy individuals and, together with the release of HMGB1, IL-6, and TNF-α, were sufficiently significant in the ROC analysis to identify the group with impaired NLRP3 inflammasome activation (Supplementary Table 4), all with an area under the curve of 0.9–1.

We next found that the septic patients with immunocompromised NLRP3 accounted for over 80% of the deaths registered in the group of septic patients during their stay at the Surgical Critical Unit (Fig. 2c, Supplementary Table 2) from day 9 onwards (Fig. 2d). Septic patients at day 1 were assessed using different biochemical and clinical scores in order to determine disease severity, but these tests were unable to accurately discriminate the group with profound NLRP3 immunosuppression (Fig. 2e, f, Supplementary Fig. 2f, g). Nevertheless, patients with severe NLRP3 suppression did present higher SOFA, but without statistical differences (Fig. 2f). There were also no differences in plasma cytokines, monocytes with active caspase-1, circulating ASC-specks (Supplementary Fig. 2h), or infection type among septic patients with profound NRLP3 immunosuppression (Supplementary Tables 2 and 5), although NLRP3-immunocompromised patients suffered more often from nosocomial infection (Supplementary Table 2). Mortality was not the only late-outcome associated with early NLRP3-immunocompromised septic patients, for example, the evolution of clinical parameters, such as ΔSOFA (calculated as the change in SOFA from day 1 to day 5) increased in these patients (Fig. 2g, Supplementary Table 4), thus confirming that ΔSOFA is an accurate late-prognostic marker for sepsis-related deaths^30^, while profound NLRP3 immunoparalysis emerged as a potential early prognostic marker. Also, NLRP3-immunocompromised septic patients presented a longer stay at the surgical critical unit and required more days of mechanic ventilation (Fig. 2h), as well as presented a non-significant increase of renal, respiratory, and cardiovascular dysfunction (Supplementary Table 2), suggesting a worst outcome for the patients that presented early severe impairment of the NLRP3 activation.

### Paralysis of NLRP3 inflammasome during sepsis is transitory

**Fig. 3:**
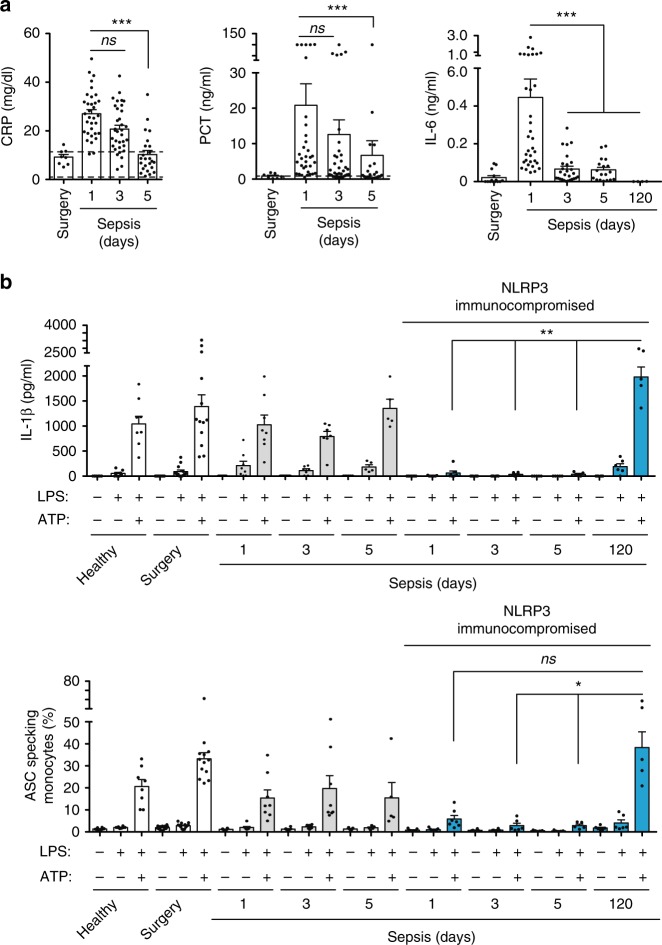
NLRP3 immunoparalysis during sepsis is transitory. **a** Concentrations of C-reactive protein (CRP), procalcitonin (PCT), and IL-6 in plasma of septic patients at days 1, 3, and 5 during sepsis and at day 120 after sepsis recovery; dotted lines represent normal concentration of CRP and PCT. Levels of these markers in septic patients were compared with the abdominal surgery controls at 24 h after surgery. Control group and septic patients at day 1 correspond to patient data presented in Fig. 1a, b, and are shown here for comparison. **b** ELISA for IL-1β in PBMC supernatants (top) and percentage of monocytes with intracellular ASC specks (bottom) after NLRP3 inflammasome activation by LPS (1 μg/ml, 2 h) and ATP (3 mM, 30 min) treatment from control groups and NLRP3 non-immunocompromised (gray) or NLRP3 immunocompromised (blue) septic patients at day 1, 3, 5 during sepsis and at day 120 after sepsis recovery. Control groups and septic patients at day 1 correspond to patient data presented in Fig. 2a, b, and are shown here for comparison; each dot represents an individual patient; average ± standard error is represented in all panels; exact *n* number for each panel is presented in Source Data file; *p < 0.05; **p < 0.01; ***p < 0.001; *ns*, no significant difference (*p* > 0.05); Kruskal–Wallis test for **a**, **b**

Plasma concentration of CRP, PCT, and IL-6 decreased after 3 and 5 days of sepsis onset (Fig. 3a), thus indicating a gradual resolution of systemic inflammation. However, severe NLRP3 immunoparalysis was present from days 1 to 5 in septic patients, in whom NLRP3 activation by extracellular ATP in PBMCs was not able to release IL-1β and whose monocytes presented impaired intracellular ASC speck formation (Fig. 3b). Normal release of IL-1β and ASC-speck formation was found in non-compromised NLRP3 septic patients during the course of the first 5 days of sepsis (Fig. 3b). Four NLRP3 compromised patients survived sepsis, and from them we were able to obtain a sample from three patients once recovered. NLRP3-immunocompromised septic patients who survived and recovered from sepsis (*n* = 3) did not have detectable levels of IL-6 in their plasma at 120 days after the septic episode (Fig. 3a, Supplementary Fig. 3a), but their NLRP3 inflammasome could be activated normally (Fig. 3b, Supplementary Fig. 3b). This suggests that NLRP3 inflammasome impairment during sepsis is transitory.

### P2X7 receptor is upregulated in monocytes during sepsis

**Fig. 4:**
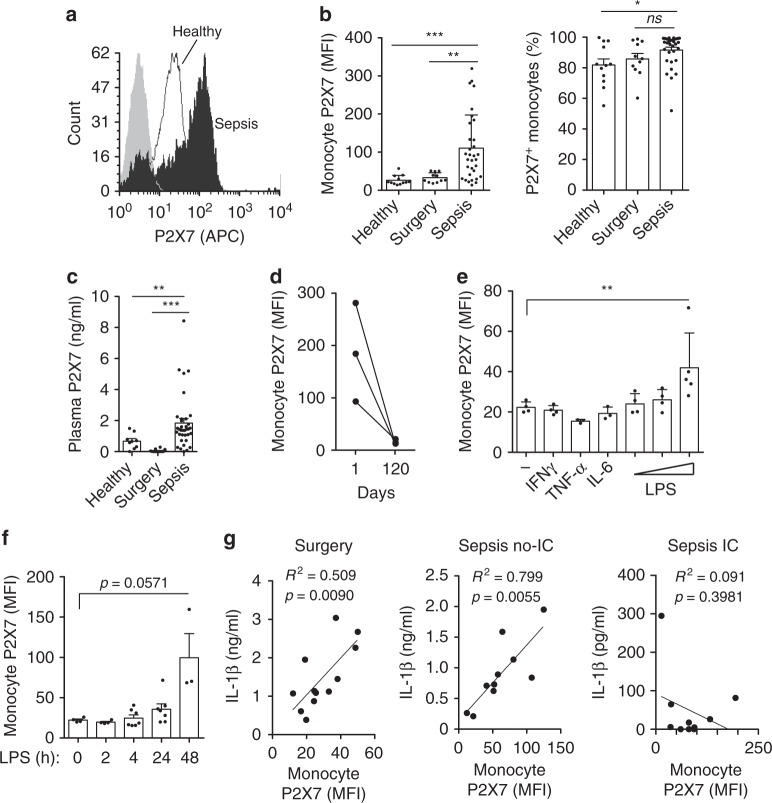
P2X7 receptor is transiently upregulated in monocytes during sepsis. **a** Representative histogram plot of surface P2X7 receptor staining in monocytes from healthy (white), septic patient (black) and non-stained monocytes (gray). **b** Quantification of P2X7 receptor mean fluorescence intensity (MFI, left) and percentage of positive monocytes for P2X7 receptor (right) in control and septic patients. **c** ELISA to quantify the concentration of soluble P2X7 receptor in plasma of control and septic patients. **d** Quantification of P2X7 receptor MFI at day 1 during sepsis and day 120 after recovery. **e** Quantification of P2X7 receptor MFI in monocytes from healthy donors treated with IFNγ, TNF-α, IL-6 (all at 20 ng/ml), or with increasing concentrations of LPS (10, 100, 1000 ng/ml) for 24 h. **f** Quantification of P2X7 receptor MFI in monocytes from healthy donors treated with LPS (1 μg/ml) for the indicated times. **g** Correlation between the concentration of IL-1β released from PBMCs treated with LPS (1 μg/ml, 2 h) and ATP (3 mM, 30 min), and the quantification of P2X7 receptor MFI in monocytes from the indicated control and septic individuals; IC: immunocompromised septic patients. Each dot represents an individual septic patient or healthy donor; average ± standard error is represented in panels **b**, **c**, **e**, **f**; exact *n* number for each panel is presented in Source Data file; *p < 0.05; **p < 0.01; ***p < 0.001; *ns*, no significant difference (*p* > 0.05); Kruskal–Wallis test was used in **b**; Mann–Whitney test for **c**, **e**, **f**; Pearson correlation was used in **g**

In order to investigate the possible causes of NLRP3 impairment during sepsis, we aimed to study the P2X7 receptor in monocytes as this is the receptor for extracellular ATP, the ligand we used to activate the inflammasome in monocytes from septic patients. We first found that the surface expression of P2X7 receptor was higher in the monocytes of septic patients than in the control groups (Fig. 4a, b), although the percentage of P2X7^+^ monocytes was similar among septic patients and control groups (Fig. 4b). This increase was also observed in the levels of soluble P2X7 receptor detected in plasma (Fig. 4c), which increased on the surface of monocytes during the 5 first days of sepsis and then reduced upon sepsis recovery (Fig. 4d, Supplementary Fig. 3c). As expected, the population of CD14^+^CD16^++^ inflammatory monocytes increased during sepsis (Supplementary Fig. 3d), but the surface expression of P2X7 receptors increased in all populations of monocytes (Supplementary Fig. 3e). The increase in surface expression of P2X7 receptors in monocytes during sepsis was similar in both NLRP3 compromised and non-compromised septic patients (Supplementary Fig. 3f). The stimulation of healthy individual monocytes with LPS, but not IL-6, TNF-α, or IFNγ, increased the surface expression of P2X7 receptors (Fig. 4e, f), suggesting that bacterial infections rather than the pro-inflammatory cytokines that are present during the initial phase of sepsis are responsible for the increase in P2X7 receptor expression observed during sepsis.

### P2X7 receptor correlates with mitochondrial depolarization

**Fig. 5:**
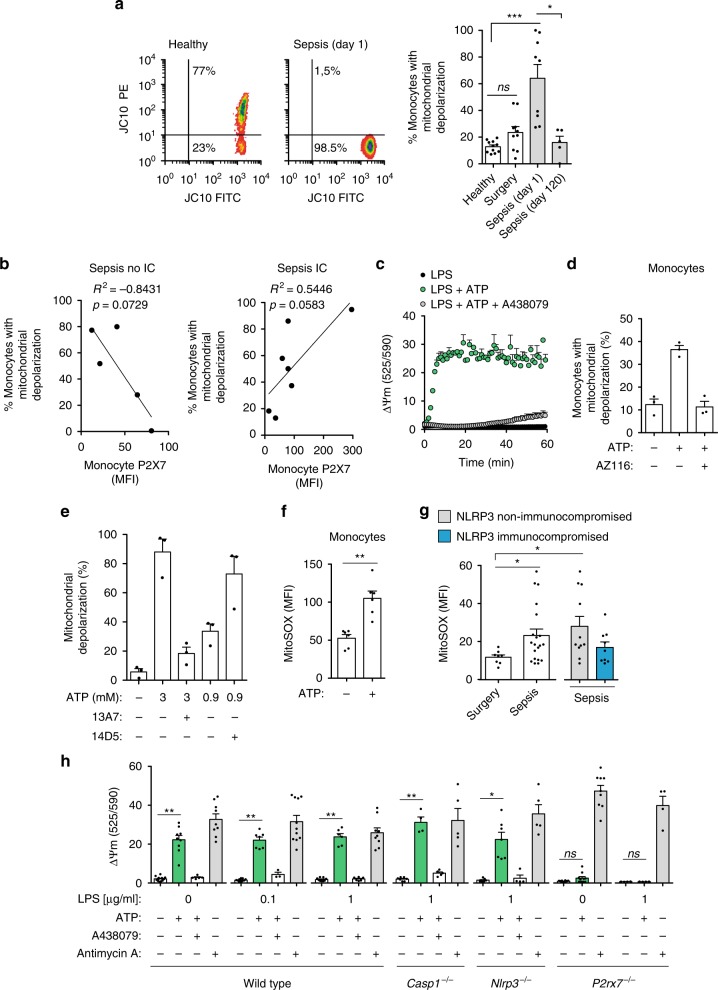
P2X7 receptor induces mitochondrial membrane depolarization. **a** Mitochondrial membrane potential from blood monocytes stained with JC-10 in a control healthy donor (left), in a septic patient at day 1 (middle), and from healthy controls, abdominal surgery, and septic patients at day 1 or 120 (right); PE^−^ cells represent the monocytes with mitochondrial depolarization. **b** Correlation between the percentage of monocytes with mitochondrial membrane depolarization and the quantification of P2X7 receptor MFI in monocytes from the septic patients; IC: immunocompromised septic patients. **c** Kinetics of mitochondrial depolarization after ATP (3 mM) stimulation of LPS-primed BMDMs incubated or not with the P2X7 receptor antagonist A438079 (10 μM). **d** Percentage of human monocytes from healthy donors with mitochondrial membrane depolarization after ATP (3 mM, 30 min) stimulation and incubated or not with the P2X7 receptor antagonist AZ11645373 (10 μM). **e** Percentage of mouse BMDMs with mitochondrial membrane depolarization after ATP stimulation at the indicated concentrations for 30 min and incubated or not with anti-P2X7 nanobodies (13A7, blocking nanobody; 14D5, potentiating nanobody; each at 200 nM). **f** Mitochondrial ROS production from human monocytes isolated from healthy donors after ATP (3 mM, 30 min) stimulation. **g** Mitochondrial ROS from human monocytes isolated from surgery controls and septic patients (white bar); right bars separate the group of septic patients into NLRP3 non-immunocompromised (gray bar) and immunocompromised (blue bar). **h** Mitochondrial membrane depolarization from wild-type or knock-out BMDMs as indicated primed or not with LPS (4 h) and treated for 30 min with ATP (3 mM, green bars), ATP and A438079 (10 μM, white bars), or antimycin A (5 μM, gray bars). Each dot represents an individual patient in **a**, **b**, **g**, or an individual healthy donor in **d**, **f**, a single independent experiment in **e**, **h**, or average + SEM of two independent experiments in **c**; average ± standard error is represented in panels **a** (right), **c**–**h**; exact *n* number for each panel is presented in Source Data file; *p < 0.05; **p < 0.01; ***p < 0.001; *ns*, no significant difference (*p* > 0.05); Pearson correlation was used in **b**; Mann–Whitney test was used in **f**; and Kruskal–Wallis test was used in **a**, **g**, **h**

We next found that P2X7 receptor expression positively correlated with the release of IL-1β after ATP stimulation in surgery control patients and non-compromised NLRP3 septic patients (Fig. 4g). However, in monocytes from NLRP3 compromised septic patients, P2X7 receptor expression did not correlated with IL-1β release (Fig. 4g), suggesting an alternative role for P2X7 receptors in sepsis. P2X7 receptors have been previously associated with mitochondrial damage and some studies indicate that defects in the energy metabolism of monocytes underlay immunoparalysis in sepsis^3,4,31–33^. In our cohort of septic patients, there was an increase in monocyte mitochondrial membrane depolarization that was reestablished when the patients recovered from sepsis (Fig. 5a). Furthermore, the P2X7 receptors in the monocytes of NLRP3-compromised septic patients positively correlated with mitochondrial depolarization, a phenomenon that was negatively correlated in non-compromised NLRP3 septic patients (Fig. 5b). P2X7 receptor stimulation in monocytes and macrophages resulted in a fast-mitochondrial membrane depolarization that was reversed using P2X7 receptor antagonists and the anti-P2X7 receptor blocking nanobody 13A7 (Fig. 5c–e). Using the 14D5 nanobody, which strengthens the response of P2X7 receptors by lowering the ATP threshold required to activate it^34^, we observed an increase in mitochondrial depolarization in response to suboptimal ATP concentrations for P2X7 receptors (Fig. 5e). Mitochondrial membrane depolarization induced by ATP was hardly affected by the ionic flow of Ca^2+^ or Na^+^ through the P2X7 receptor (Supplementary Fig. 3g). Mitochondrial membrane depolarization induced by ATP was matched by an increase in mitochondrial ROS production in the monocytes (Fig. 5f, Supplementary Fig. 3h) and was higher in septic patients than in the control surgery group (Fig. 5g). Mitochondrial depolarization induced by ATP occurred independently of LPS-priming and the NLRP3 inflammasome (Fig. 5h). Mitochondrial depolarization was also confirmed using the green MitoTracker mitochondrial dye, which stains to a greater or lesser extent depending on the mitochondrial membrane potential (Supplementary Fig. 4a). However, this change on the mitochondrial membrane potential did not damage the mitochondria integrity, as is demonstrated by the absence of cytochrome c in the cytosol fraction after P2X7 receptor activation (Supplementary Fig. 4b). This was further confirmed by electron microscopy, which showed no damage to the mitochondria when ATP was applied, and instead revealed a swelling of the mitochondria that was dependent on P2X7 receptor activation, this was evidenced by the use of a specific P2X7 receptor antagonist that reversed it (Supplementary Fig. 4c). This mitochondrial swelling induced after P2X7 receptor activation was also observed by staining with the mitochondria marker Tomm20 (Supplementary Fig. 4d). These data suggest that the activation of P2X7 receptors in resting monocytes and macrophages results in mitochondrial depolarization, swelling, and ROS production, but not in fragmentation or damage of the mitochondrial structure.

### P2X7 receptors impairs NLRP3 inflammasome activation

**Fig. 6:**
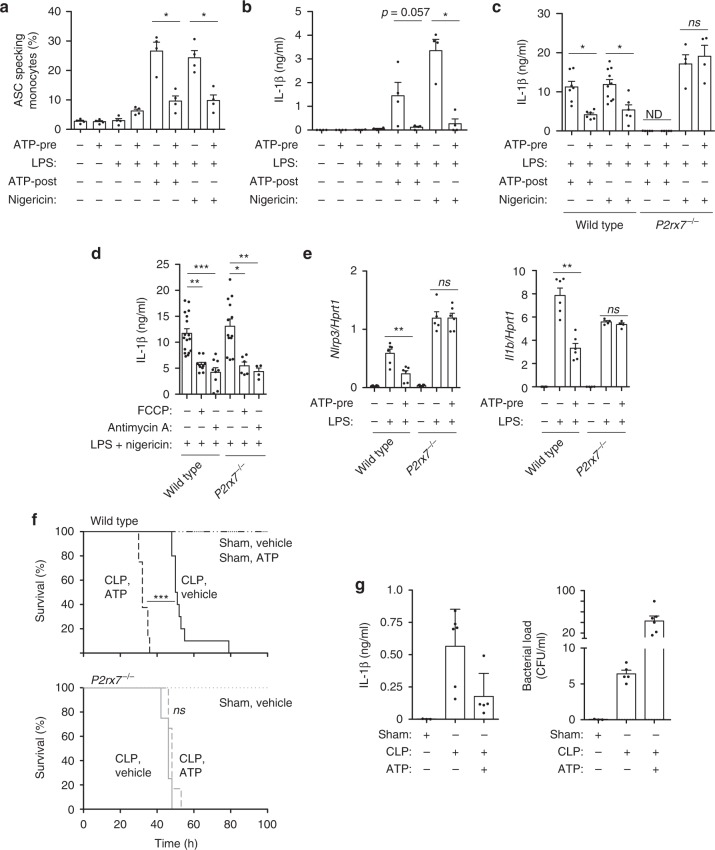
P2X7 receptor stimulation impairs the NLRP3 inflammasome in monocytes. **a**, **b** Percentage of monocytes with ASC-specks (**a**) and release of IL-1β from PBMCs (**b**) isolated from healthy donor blood samples treated with ATP (3 mM, 30 min; ATP-pre), then washed and primed with or without LPS (1 μg/ml, 2 h) and then treated for 20 min with ATP (3 mM; ATP-post) or nigericin (10 μM) as indicated. **c** IL-1β release from wild type or *P2rx7*^−/−^ BMDMs treated as in **a**, but with 4 h LPS priming and 30 min of ATP or nigericin. **d** IL-1β release from wild type or *P2rx7*^−/−^ BMDMs treated for 30 min with antimycin A (5 μM) or FCCP (1 μM), and then washed and primed with LPS (1 μg/ml, 4 h) and then stimulated with nigericin (10 μM, 30 min) as indicated. **e** Expression of *Nrlp3* and *Il1b* genes analyzed by quantitative PCR from wild type or *P2rx7*^−/−^ BMDMs treated with ATP (3 mM, 30 min; ATP-pre), then washed and primed with or without LPS (100 ng/ml, 4 h). **f** Kaplan–Meier representation of wild type (top) or *P2rx7*^−/−^ (bottom) mice survival after sham operation (dotted line), or CLP operation (continuous line); some mice groups were i.p. injected with ATP (0.5 mg/g) 30 min before operation (dashed lines). Wild type CLP *n* = 10, CLP + ATP *n* = 8, sham *n* = 4, sham + ATP *n* = 4; *P2rx7*^−/−^ CLP *n* = 4, CLP + ATP *n* = 6, sham *n* = 3. **g** IL-1β from peritoneal lavage (left) or bacterial load in blood (right) from wild type sham, CLP or CLP+ATP mice after 24 h. Each dot represents a single independent experiment (**c**, **d**), a sample from an individual healthy donor (**a**, **b**, **e**) or a single mouse (**g**); average ± standard error is represented in panels **a**–**e**, **g**; exact *n* number for each panel is presented in Source Data file; *p < 0.05; **p < 0.01; *ns*, no significant difference (*p* > 0.05); Mann–Whitney test was used for **a**, **b**, **d**, **e**; Kruskal–Wallis test was used for **c**; Log-rank test for **f**

Having found that stimulating P2X7 receptors in unprimed monocytes and macrophages induced mitochondrial membrane depolarization, we then found simultaneously that NLRP3 inflammasome activation was impaired after LPS-priming and subsequent ATP or nigericin treatment (Fig. 6a–c). Stimulation of the P2X7 receptor before NLRP3 priming and activation decreased the formation of intracellular ASC specks (Fig. 6a) and impaired the release of IL-1β (Fig. 6b, c). This effect was reverted using a specific antagonist for P2X7 receptor (Supplementary Fig. 5a). P2X7-receptor-deficient macrophages did not present NLRP3 inflammasome inhibition when ATP was applied before LPS priming (Fig. 6c), suggesting that activation of P2X7 receptor before LPS priming decrease NLRP3 inflammasome activation. NLRP3 impairment was independent of K^+^-efflux through the P2X7 receptor (Supplementary Fig. 5b). This result was similar to the response of monocytes isolated from profoundly NLRP3-immunocompromised septic patients, in whom P2X7 receptor expression correlated with mitochondrial dysfunction (Fig. 5b). IL-1β release was also reduced when mitochondrial membrane depolarization was induced by FCCP or antimycin A, and this effect was independent of the P2X7 receptors, given that P2X7-receptor-deficient macrophages also released less IL-1β in response to FCCP or antimycin A (Fig. 6d, Supplementary Fig. 5c). ATP, FCCP, and antimycin A treatment did not induce cell death (Supplementary Fig. 5d, e). Gene expression for *Nlrp3* and *Il1b* was partially reduced when the P2X7 receptors were activated before LPS priming in wild type mice, but not in P2X7-receptor-deficient macrophages (Fig. 6e), suggesting that P2X7 activation could also induce a possible defect in inflammasome priming. Similarly, mitochondrial membrane depolarization induced by antimycin A reduced *Nlrp3* and *Il1b* gene induction by LPS (Supplementary Fig. 5f).

To determine if P2X7-receptor activation before bacterial priming was had a significant effect on decreasing survival rates during sepsis, we performed cecal ligation and puncture (CLP) in wild type and *P2rx7*^−/−^ mice with an initial i.p. ATP injection. We found a significant reduction in the survival of *P2rx7*^−/−^ mice compared to wild type animals after CLP (Supplementary Fig. 5g), which is consistent with previous studies^21,22^. Injection of ATP before CLP significantly decreased the survival rates of wild type mice, but not of *P2rx7*^−/−^ mice (Fig. 6f). P2X7 receptor activation in vivo before infection decreased the release of IL-1β into the mouse peritoneum (Fig. 6g) and prevented effective control of the infection as the bacterial load in the blood increased in animals pre-treated with ATP (Fig. 6g).

**Fig. 7:**
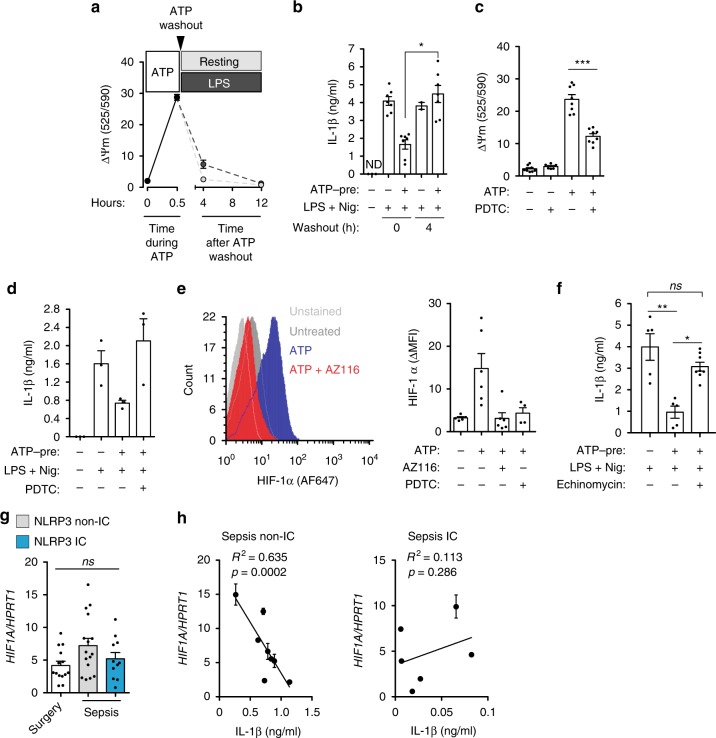
Mitochondrial dysfunction mediates P2X7 receptor-induced NLRP3 inflammasome impairment. **a** Mitochondrial membrane depolarization in BMDMs treated with ATP (3 mM, 30 min), then washed-out and incubated for the indicated times with or without LPS (1 μg/ml). **b** IL-1β release from wild-type BMDMs treated as in **a**, but after LPS priming cells were stimulated with nigericin (10 μM, 30 min). **c** Mitochondrial membrane depolarization in BMDMs treated with ATP (3 mM, 30 min) in the presence or absence pyrrolidine dithiocarbamate (PDTC, 40 μM). **d** IL-1β release from PBMC isolated from healthy donor blood samples treated with ATP (1 mM, 30 min; ATP-pre) with or without PDTC (10 μM), then washed and primed with or without LPS (1 μg/ml, 4 h) and then stimulated with nigericin (10 μM, 30 min). **e** Representative histogram plot of HIF-1α staining (left) or quantification of mean intensity fluorescence increase (ΔMFI, right) in monocytes from healthy donor blood samples treated or not with ATP (1 mM, 30 min) in the presence or absence of AZ11645373 (10 μM) or PDTC (10 μM), and then washed and cultured for 2 h; non-stained monocytes (light gray, left). **f** IL-1β release from BMDM supernatants treated with ATP (3 mM, 30 min; ATP-pre) in the presence or absence of echinomycin (5 nM), then washed and primed with or without LPS (1 μg/ml, 4 h) and then stimulated with nigericin (10 μM, 30 min). **g** Expression of *HIF1A* analyzed by qPCR from control surgery group and septic patients PBMCs; septic patients are separated into NLRP3 non-immunocompromised (gray bar) and immunocompromised (blue bar). **h** Correlation between *HIF1A* expression and IL-1β released from septic patients PBMCs treated with LPS (1 μg/ml, 2 h) and ATP (3 mM, 30 min); IC: immunocompromised septic patients. Each dot represents a single independent experiment or a sample from an individual healthy donor or septic patient; average ± standard error is represented in panels **a**–**g**; exact *n* number for each panel is presented in Source Data file; *p < 0.05; **p < 0.01; ***p < 0.001; *ns*, no significant difference (*p* > 0.05); Kruskal–Wallis test was used for **b**, **c**, **f**; Pearson correlation was used in **h**

NLRP3 impairment induced by P2X7 receptor activation in cultured macrophages was transitory, and mitochondrial membrane potential was restored after washing extracellular ATP for 4–12 h (Fig. 7a), as was the ability of macrophages to produce IL-1β normally after NLRP3 activation (Fig. 7b). The antioxidant pyrrolidine dithiocarbamate (PDTC) was able to protect against ATP-induced mitochondrial-membrane depolarization (Fig. 7c) and restored the production of IL-1β after stimulating the P2X7 receptors with ATP before LPS-priming and NLRP3 activation (Fig. 7d), thus suggesting that NLRP3 impairment induced by P2X7-receptor activation could be mediated by mitochondrial dysfunction. Given that hypoxia-inducible factor (HIF)-1α has been described as an important factor for the metabolic reprogramming of myeloid cells during sepsis, we next analyzed how P2X7 receptors could be controlling HIF-1α. We found that P2X7 receptor activation by ATP increased HIF-1α expression in human monocytes (Fig. 7e). However, FCCP and antimycin A did not increase HIF-1α (Supplementary Fig. 5h), which suggests that the P2X7 receptor action was differentially rather than directly targeting complex III or directly transporting protons across mitochondrial inner membrane. HIF-1α expression induced by ATP treatment was dependent on the P2X7 receptors and mitochondrial membrane depolarization, since P2X7 antagonist AZ116 and PDTC treatment during ATP stimulation were both able to prevent it (Fig. 7e). Blocking HIF-1α with echinomycin restored the production of IL-1β after the P2X7 receptors were stimulated with ATP before LPS-priming and NLRP3 stimulation (Fig. 7f). HIF-1α was expressed in septic patients and in the surgery control group (Fig. 7g), and the higher HIF-1α expression in NLRP3 non-immunocompromised patients correlated with a lower IL-1β release (Fig. 7h). In conclusion, our data support a model in which the P2X7 receptors affect mitochondria and impair NLRP3 inflammasome in monocytes, a process that could contribute to immunosuppression in septic patients.

## Discussion

Sepsis remains the leading cause of death in critical care units^35^. Our study reveals that during the initial inflammatory response in sepsis, septic patients present an early impairment of the NLRP3 inflammasome that is associated with higher mortality. The expression of P2X7 receptors increased in monocytes from septic patients and correlated with mitochondrial membrane dysfunction, and not with the secretion of IL-1β. Mechanistically, P2X7 receptor activation in monocytes before microbial-stimulation resulted in mitochondrial damage, HIF-1α expression, and impairment of the NLRP3 inflammasome activation.

Sepsis causes a systemic inflammatory response driven by the production of proinflammatory cytokines. IL-6 and IL-18 have been proposed as potential biomarkers for septic patients^24,36–39^. Our study demonstrates that at day 1, the inflammasome-related cytokines IL-1β and IL-18, the alarmin HMGB1 and circulating aggregates of ASC are higher in the blood of septic patients of intra-abdominal origin, which coincides with previous publications that found elevated inflammasome gene expression in monocytes and circulating IL-18 during sepsis^24,29^. However, while IL-18 concentration has been positively associated with mortality in sepsis^24^, IL-18 measured in our cohort of septic patients did not correlate with mortality. Also, our study did not find a significant increase in inflammasome genes in the PBMCs, in contrast with other studies that have found an increase in *NLRP3*, *CASP1*, and *PYCARD*^16,28^. These differences could be due to the inclusion of patients with lung infections^16^ or to different ranges of etiologies among the septic patients enrolled in other studies^24,28^, in contrast to our study, which focuses on a well-defined population of intra-abdominal septic patients. Different studies have demonstrated that human blood monocytes from septic patients responded differently to ex vivo bacterial endotoxin challenge^4,40–45^, and only one study has found an impaired inflammasome response in septic patients^17^. The elevated risk of late-deaths in sepsis is thought to be related to the immunosuppressive state of leukocytes in these patients^2,41,46^. In the present study, we report a differential activation of the NLRP3 inflammasome in septic patients, and have found that patients with profound NLRP3 deactivation accounted for most late deaths. These patients also presented impaired production of other cytokines when PBMCs were stimulated ex vivo, suggesting a general suppression of innate immunity. Although the NLRP3-immunocompromised septic cohort that we studied is relatively small, NLRP3 function accurately identified patient death over other early clinical scores. Impairment of NLRP3 inflammasome activation was found as early as within the first 24 h after patient enrollment in our study, when there were high levels of inflammatory and inflammasome markers in the blood. This supports previous data demonstrating that the release of IL-1β is compromised in ex vivo LPS-stimulated PBMCs collected after 2 h of intravenous LPS infusion in healthy volunteers^17^, and that within 24 h of the onset of human sepsis NLRP1 gene expression decrease in monocytes^47^. Therefore, NLRP3 inflammasome deactivation in sepsis overlap a systemic proinflammatory response, which supports the idea that in critically injured patients pro-inflammatory and anti-inflammatory responses could coexist at the same time^48^.

Blood monocytes are important cells in the development of an immunocompromised state in sepsis because they not only acquire a deactivation phenotype but are also involved in suppressing lymphocyte function^49,50^. The immunocompromised state of these cells has been found to be due to defects in the metabolism of septic monocytes, including important paralysis of mitochondria with a parallel reduction of oxygen consumption^3,4,31,32^. Furthermore, the mitochondria of myeloid cells undergo adaptations in the respiratory chain upon sensing bacteria^51^, and HIF-1α appears as a key factor for the metabolic reprogramming of myeloid cells during sepsis^6,44,52,53^. Here we confirm that monocytes from septic patients present dysfunctional mitochondria, as is evidenced by the depolarization of the mitochondrial membrane, but without any structural damage. It is known that the purinergic P2X7 receptor, a receptor for extracellular ATP and a strong activator of NLRP3 inflammasome, also stimulates the induction of HIF-1α in different non-myeloid cells^12,54–56^, and we confirm this in human monocytes in the present study. We found elevated P2X7 receptor expression in the surface of monocytes from septic patients when compared to monocytes from control groups, and P2X7 receptor expression in septic patients’ monocytes was correlated with increased mitochondrial dysfunction. This confirms previous data in recombinant HEK293 cells where activation of the P2X7 receptor and subsequent increase of intracellular Ca^2+^ induce rapid mitochondrial depolarization and swelling^33^. However, in our study mitochondrial depolarization in macrophages was slightly dependent on increased intracellular Ca^2+^, suggesting that other P2X7 receptor signaling in this cell type may lead to mitochondrial depolarization. This effect was independent of LPS-priming and NLRP3 inflammasome, thus ruling out possible mitochondrial damage as a consequence of caspase-1 activation^57^. When P2X7 receptors were triggered in resting monocytes and macrophages, it decreased the response of these cells to further activate the NLRP3 inflammasome, thus inducing a deactivation state in the monocyte similar to the state found in immunocompromised septic patients. This was also supported by a mouse model of sepsis, in which P2X7-receptor-deficient mice had a lower survival rate than wild type animals, thus corroborating previous work using the CLP model or i.v. injection of *E. coli*^21,22^. We also found that activation of P2X7 receptors in vivo before sepsis increased mouse mortality, which is similar to previous observations regarding LPS-induced mortality^19^. However, one previous study found that the P2X7-deficient mice present increased survival rates after the CLP model^20^. This controversy not only demonstrates the variability of the CLP model^23^, but also highlights the fact that sepsis survival is a fine balance between the degree of inflammasome-activation and downstream cytokine production. This process is critically modulated by the time of P2X7 receptor activation, and this may lead to the repression or induction of the NLRP3 inflammasome. This effect was dependent on HIF-1α after mitochondrial dysfunction in monocytes and macrophages when analyzed in vitro. In this situation, P2X7 receptor activation prevents and does not induces, NLRP3 inflammasome activation. Similarly, it is known that in M2 macrophages ATP is able to prevent NLRP3 inflammasome activation, but this effect was independent of P2X7 receptors^58^. An increase in extracellular ATP concentration due to complications during surgery or as consequence of different treatments^59^, could activate P2X7 receptors before or during the early phase of an infection, and might contribute to the development of immunosuppression by impairing the NLRP3 inflammasome.

In conclusion, we found that activation of NLRP3 inflammasome in monocytes is compromised in septic patients, where P2X7 receptor expression is associated with mitochondrial dysfunction but not with IL-1β release. Activation of P2X7 receptors in resting myeloid cells before priming with microbial-associated molecules impaired NLRP3 inflammasome activation via mitochondrial damage and HIF-1α production. Restoration of P2X7 receptor expression levels and NLRP3 inflammasome activation in monocytes could be a good indicator of immune recovery in septic patients. Therapies aiming to decrease extracellular ATP or to block the P2X7 receptor at early time points, could help provide individualized treatment for septic patients and improve survival rates among patients.

## Methods

### Human clinical samples

The clinical ethics committee of the Clinical University Hospital *Virgen de la Arrixaca* (Murcia, Spain) approved this study and its procedures (reference number PI13/00174). The samples and data from patients included in this study were provided by the *Biobanco en Red de la Región de Murcia* (PT13/0010/0018), which is integrated into the Spanish National Biobanks Network (B.000859). All study procedures were conducted in accordance with the declaration of Helsinki. Whole peripheral blood samples were collected after receiving written informed consent from intraabdominal sepsis patients (*n* = 35, Supplementary Table 1) at the Surgical Critical Unit from the Clinical University Hospital *Virgen de la Arrixaca* (Murcia, Spain) after 1, 3, 5, and 120 days of sepsis development, day 1 being the blood sample obtained within 24 h of the diagnosis of sepsis. Acute physiology and chronic health evaluation II (APACHE II) and SOFA, different clinical, microbiological, hemodynamic, and biochemical determinations were routinely evaluated in all septic patients at different days by the Clinical University Hospital *Virgen de la Arrixaca* Surgical Critical Unit, Clinical Analysis and Microbiology Units. All the septic patients included in this study met the definition for severe sepsis or septic shock that was valid at the time of sample and data collection^60^. The inclusion criteria for septic patients were patients diagnosed with intra-abdominal origin sepsis confirmed by exploratory laparotomy, with at least two diagnostic criteria for sepsis (fever or hypothermia; heart rate >90 beats per minute; tachypnea, leukocytosis, or leukopenia) and multiple organ dysfunction defined as physiological dysfunction in two or more organs or organ systems^60^. We excluded patients who were immunocompromised or presented immunodeficiency (including antineoplastic treatments during the month previous to the septic episode). We also excluded terminal oncologic and hematologic neoplastic patients, as well as patients that had a delay of >24 h from intra-abdominal sepsis diagnosis to surgery, patients who spent <24 h in the Surgical Critical Unit, those whose infection was not cleared by the surgery and patients who presented another septic focus different from the abdominal focus. We also analyzed whole peripheral blood samples from healthy volunteers (*n* = 11) and abdominal surgery patients, who had not developed sepsis (*n* = 14, Supplementary Table 1).

### CLP model

All animal work was in accordance with Spanish national (RD 53/2013) and EU (86/609/EEC and 2010/63/EU) legislation. The University of Murcia Animal Research Ethical Committee approved animal procedures (ref. 5/2014) and then the Animal Health Service of the General Directorate of Fishing and Farming of the Council of Murcia (*S*er*vicio de Sanidad Animal, Dirección General de Ganadería y Pesca, Consejería de Agricultura y Agua Región de Murcia*) approved animal procedures with ref. A1320140201. CLP-induced sepsis was performed in C57BL/6 (WT, wild-type) and P2X7R-deficient (*P2rx7*^*−*/−^) mice in C57BL/6 background. Thirty minutes before CLP, a group of mice received an intraperitoneal injection of ATP (0.5 mg/g) or saline vehicle. Laparotomy was performed to isolate the cecum of mice anesthetized with isoflurane. Approximately 2/3 of the cecum was ligated with a 6–0 silk suture and punctured twice through-and-through with a 21 gauge needle. The abdominal wall and incision were then closed with 6–0 silk suture. Sham-operated animals underwent laparotomy without ligation or puncture of the cecum. Buprenorphine (0.3 mg/kg) was administered intraperitoneally at the time of surgery and mice were monitored continuously until recovery from anesthesia. For sample collection, 24 h after the procedure, animals were euthanized with CO_2_ inhalation, and peritoneal lavages were performed with 4 ml of sterile saline and then blood was collected from the thoracic aorta. Serum and centrifuged (cell free) peritoneal lavages were stored at −80 °C until further analysis. Serum was diluted serially in sterile physiologic saline and plated and cultured on agar plates at 37 °C for 24 h. Then the number of bacterial colonies was counted and expressed as CFU/ml of serum.

### Cells and treatments

Human PBMCs were isolated from blood within one hour after extraction using Ficoll histopaque 1077 (Sigma-Aldrich). BMDM were obtained from wild-type, *Nlrp3*^−/−^, *Casp1/11*^−/−^, and *P2rx7*^−/−^ mice by differentiating bone marrow cells for 7 days in DMEM (Lonza) supplemented with 25% of L929 medium, 15% fetal calf serum (FCS, Life Technologies), 100 U/ml penicillin/streptomycin (Lonza), and 1% l-glutamine (Lonza)^61^. THP-1 cells were maintained in RPMI1640 media with 10% FCS and 2 mM GlutaMAX (Life Technologies). Cells were treated either in tissue culture plates or directly in flow cytometry tubes with ATP (Sigma-Aldrich), antimycin A (Sigma-Aldrich), or FCCP (Sigma-Aldrich) in the presence or absence of PDTC (Sigma-Aldrich) or echinomycin (Sigma-Aldrich) in E-total buffer (147 mM NaCl, 10 mM HEPES, 13 mM glucose, 2 mM CaCl_2_, 1 mM MgCl_2_, and 2 mM KCl, pH 7.4) and then washed and stimulated with *E. coli* LPS O55:B5 in their respective complete media. In some experiments, PBMCs were treated with recombinant human IL-6, TNF-α, or IFNγ (PeproTech). After LPS treatment, cells were incubated with P2X7-modulating nanobodies^34^, the specific P2X7 receptor antagonist A438079 or AZ11645373 (Tocris), and then subsequently stimulated with ATP or nigericin (Sigma-Aldrich) in E-total buffer for 20–30 min as specified in the figure legends. Times and concentrations for the reagents used are specified in the figure legends. 23 out of the 35 septic patients included in this study (65.7%) were able to provide enough blood samples for in vitro PBMC stimulation.

### ELISA and multiplexing assay

Cytokines and soluble P2X7 in plasma were measured by ELISAs from eBioscience, IBL International, R&D Systems, Cusabio (for soluble P2X7), and MBL following the manufacturers’ indications and read in a Synergy Mx (BioTek) plate reader. Multiplexing was performed using the Cytometric Bead Array from Becton Dickinson Biosciences following the manufacturer indications, and were analyzed in a BD FACS Canto.

### Flow cytometry

Intracellular ASC-speck formation was evaluated by the Time of Flight Inflammasome Evaluation in CD14^+^ monocytes using a polyclonal unconjugated rabbit anti-ASC (N-15)-R antibody (catalog sc-22514-R, SantaCruz Biotechnology), and a secondary monoclonal donkey anti-rabbit antibody Alexa Fluor-488 (catalog A21206, Life Technologies), both at 1:1000 dilution. HIF-1α expression was detected in CD14^+^ monocytes using Alexa Fluor-647 mouse anti-human HIF-1α (BD Biosciences, Clone 54, catalog 565924) at 10 μl per 5 × 10^5^ cells, using a transcription factor buffer set (BD Biosciences) following manufacturer’s instructions. Cells treated in the same conditions and without staining for HIF-1α were used as a background control. Monocytes were determined from PBMCs by CD3^−^ CD14^+^ selection, and P2X7 receptor surface expression was determined in CD16^−/+/++^ monocytes using the monoclonal anti-P2X7 L4 clone conjugated with APC^62^. Active caspase-1 was measured in monocytes using the specific fluorescent probe FLICA-660 Caspase-1 Assay Kit (Immunochemistry Technologies) following the manufacturer’s instructions. Production of ROS was measured in monocytes using the red mitochondrial superoxide indicator MitoSOX (Life Technologies) following the manufacturer’s instructions. For gating strategy used in flow cytometry see Supplementary Figs. 6 and 7. The detection of extracellular particles of ASC was performed on 0.5 ml of human plasma by flow cytometry using a polyclonal unconjugated rabbit anti-ASC (AL177) antibody (catalog AG-25B-0006-C100, Adipogen) at 1:1000 dilution, and a secondary Alexa Fluor 647F(ab′) fragment of goat anti-rabbit IgG (H+L) (catalog A21246, Life Technologies) at 1:2000 dilution^14^. For the determination of ASC specks, HIF-1α expression, and FLICA, monocytes were selected using anti-CD14 PE (clone 61D3, catalog 50-0149-T025, Tonbo biosciencies) or anti-CD14 APC-H7 (clone MφP9, catalog 560180, BD Biosciences). For MitoSOX determination monocytes were selected using anti-CD33 APC-vio770 (clone REA775, catalog 130-111-139, Miltenyi). Samples were analyzed by flow cytometry using FACS Canto (BD Biosciences) and the FCS express software (De Novo Software). For multiparametric flow cytometry experiments a fluorescence minus one (FMO) control was included following recommendation of the recent flow cytometry guides for immunological studies^63^.

### Mitochondrial membrane potential

Mitochondrial membrane potential was measured using the JC-10 dye (Abcam) in BMDMs with a Sinergy Mx plate reader (BioTek) or by flow cytometry using the BD FACS Celesta flow cytometer (BD Biosciences), or in human monocytes (CD14^+^ labeled with anti-CD14 APC-H7, clone MφP9, catalog 560180, BD Biosciences) using FACS Canto (BD Biosciences), following the indications of manufacturer.

### LDH assay

Presence of LDH in cell-free supernatants was measured using the Cytotoxicity Detection kit (Roche) following the manufacturer’s instructions.

### Quantitative reverse transcriptase-PCR analysis

Total RNA was extracted from cells using the RNeasy Mini kit (Qiagen), followed by reverse transcription using iScript cDNA Synthesis (Bio-Rad). The mix SYBR Premix ExTaq (Takara) was used for quantitative PCR in iCycler MyiQ thermocycler (Bio-Rad). Specific validated primers for quantitative PCR were purchased from Sigma-Aldrich^61^. Relative gene expression levels were calculated using the 2^−ΔCt^ method normalizing to *Hprt1* expression as endogenous control.

### Statistical analysis

Statistics were calculated with Prism software (GraphPad Software Inc.). Normality of the samples was determined with D’Agostino and Pearson omnibus K2 normality test and samples did not follow a Gaussian distribution. Outliers from data sets were identified by the ROUT method with *Q* = 1%. Non-parametric Mann–Whitney test was used to analyze differences between two non-paired groups, Wilcoxon test was used to compare two paired groups, and Kruskal–Wallis test was used to analyze differences among three or more groups. The *χ*^2^-test was used to determine whether there was a significant difference between different clinical variables among groups of septic patients. Kaplan–Meier was used to estimate the survival of septic patients and the log-rank test was used to compare the survival distributions of samples.

### Reporting summary

Further information on research design is available in the Nature Research Reporting Summary linked to this article.

## Supplementary information

Supplementary Information

Reporting Summary

Source Data

## Data Availability

Data used to construct Figs. 1–7 are presented in the Source Data file. All other data are available from the corresponding author upon reasonable request.
