# Supplementary material for: P2X7 receptor induces mitochondrial failure in monocytes and compromises NLRP3 inflammasome activation during sepsis
Source: Nat Commun. 2019 Jun 20;10:2711. doi: 10.1038/s41467-019-10626-x (PMC6586640; doi:10.1038/s41467-019-10626-x)
Supplement: Supplementary file 1 — Supplementary Information [file 41467_2019_10626_MOESM1_ESM.pdf]

P2X7 receptor induces mitochondrial failure in monocytes and compromise NLRP3 inflammasome activation during human sepsis

Martínez-García et al.

SUPPLEMENTARY METHODS:

Determination of cytochrome c in cytosol

To determine cytochrome c release from mitochondria, 2 x10^6 cells per sample were primed with LPS and were exposed to different stimuli for various times or left untreated as indicated in figure legends. Cells were lysed with digitonin (17.5 μg/ml)/sucrose (500 mM) solution under vigorous vortexing for 30 s. Cytosolic fractions were isolated from organelles and cell debris by centrifugation at 14,000*g* for 60 s at 4°C[1]. As reference for total cytochrome c content of the cells, organelle-comprising fractions of untreated cells were lysed in lysis buffer (50 mM Tris-HCl pH8.0, 150 mM NaCl, 2% Triton X-100) supplemented with protease inhibitor mixture (Sigma-Aldrich) for 30 min on ice and then separated from cell debris by centrifugation at 16,000*g* for 15 min at 4 °C. Samples were resolved in 4–12% precast Criterion polyacrylamide gels (Biorad) and transferred to nitrocelulose membranes (Biorad) by electroblotting[2]. Membranes were probed with different antibodies: anti-cytochrome c mouse monoclonal (1:1000 dilution, 7H8.2C12, catalogue ab13575, Abcam), anti-alpha tubulin rabbit polyclonal (1:5000 dilution, catalogue ab4074, Abcam), and anti-caspase-1 p10 rabbit polyclonal (1:1000 dilution, M-20, catalogue sc-514, Santa Cruz).

Immunofluorescence microscopy

BMDMs were seeded on coverslips. After treatment, the cells were fixed for 15 min at room temperature with 4% formaldehyde in PBS, and were then washed with PBS. Cells were blocked with 0.5% bovine serum albumin (Sigma-Aldrich) and permeabilized for 40 min at room temperature with 0.1% TritonX100 (Sigma-Aldrich) in PBS before incubation for 2 h at room temperature with anti-Tomm20 rabbit monoclonal antibody (1:200 dilution; EPR15581-54, catalogue ab186735, Abcam). Cells were washed with PBS and were then incubated for 1 h at room temperature with the appropriate fluorescence-conjugated secondary antibody (1:800 dilution; Alexa Fluor 488 donkey anti-rabbit, catalogue A21206, Life Technology), then rinsed in PBS and incubated for 10 min with DAPI (1 μg/ml). All coverslips were mounted on slides with Fluorescence mountain solution (DAKO). Images were acquired with a Nikon Eclipse *Ti* microscope equipped with ×20 S Plan Fluor objective (numerical aperture,0.45), a ×40 S Plan Fluor objective (numerical aperture, 0.6), a ×60 /0.60S Plan Fluor objective and a digital Sight DS-QiMc camera (Nikon) with a Z optical spacing of 0.4 µm and 387-nm/447-nm, 472-nm/520-nm, 543-nm/593-nm and 650-nm/668-nm filter sets (Semrock). Maximum-intensity projection of images was achieved with NIS-Elements AR software (Nikon) and ImageJsoftware (US National Institutes of Health).

Live cell imaging

BMDMs from each genotype were plated (50×10^3 cells) on a Cell Imaging Dish 170 μm, 35x10 mm (Eppendorf). On the following day, the cells were incubated with 100 nM MitoTracker Green FM (Thermo Fisher Scientific) for 30 min at 37 ºC and washed once with physiological buffer before live cell imaging analyses. The BMDMs were imaged with a Nikon Eclipse *Ti* microscope

equipped with a ×60 /0.60S Plan Fluor objective and a digital Sight DS-QiMc camera with a Z optical spacing of 0.4 μm and 472-nm/520-nm filter set. Maximum-intensity projection of images was achieved with NIS-Elements AR software (Nikon) and ImageJ software (US National Institutes of Health).

Electron microscopy

Human monocytes stimulated in suspension were centrifuged and pellets were fixed for 1.5 h at 4°C in 2.5% buffered glutaraldehyde (Sigma-Aldrich) in 0.1 M cacodylate buffer. After fixation, pellets were immersed for 8 h in 0.01 M cacodylate buffer solution, then were post-fixed for 2 h in the dark in 1% osmium tetroxide (Sigma-Aldrich) in 0.1 M cacodylate buffer. The pellets were then dehydrated in ethanol at an increasing gradient (30%, 50%, 70%, 90% and 100%), immersed for 30 min in propylene oxide (Sigma-Aldrich) and then immersed for 16 h in a mixture of propylene oxide and Epon812 resin (1:1 ratio; Tousimis), 45 min in a mixture of propylene oxide and Epon812 resin (2:1 ratio), 2h in a mixture of propylene oxide and Epon812 resin (1:1 ratio), and 2 h in a mixture of propylene oxide and Epon812 resin (1:2 ratio). Finally, the solution was exchanged for pure Epon812 solution, and the pellets were immersed overnight. The pellets were then placed in capsules, embedded in fresh Epon812 and incubated for 72 h at 70 °C. Ultrathin sections (50-70 nm in thickness) were obtained from specimens with a microtome (Leica) and were placed onto copper grids (Sigma-Aldrich). Grids were finally post-stained by immersion for 10 min in 2% uranyl acetate in veronal buffer (Lonza) and immersion for 1 min in lead citrate (SPI). Grids were then visualized with a Jeol JEM-1011 transmission electron microscope.

SUPPLEMENTARY TABLES:

Supplementary Table 1. Demographics and clinical characteristics of enrolled patients with intra-abdominal origin sepsis and control groups. Related to Figure 1.

	**Healthy controls**	**Abdominal surgery controls**	**Intra-abdominal origin septic patients**
**N**	11	14	35
**Age**, mean (range) ± SD<br><br>*p* value *vs* septic group	67.45 (49-79) ±10.68<br><br>*p*> 0.05^{ns}	66,29 (38-94) ± 16,03<br><br>*p*> 0.05^{ns}	69.33 (43-91) ±13.34
**Gender**, N (%)<br>Male<br>Female<br><br>*p* value *vs* septic group	 5 (45%)<br>6 (55%)<br><br>*p*> 0.05^{ns}	 8 (57%)<br>6 (43%)<br><br>*p*> 0.05^{ns}	 21 (60%)<br>14 (40%)
**Clinical data**<br>(*only for septic patients*)			
**Anatomical location of the initial focus**, N (%)<br>*Stomach*<br>*Duodenum*<br>*Gallbladder*<br>*Liver*<br>*Small intestine*<br>*Cecum*<br>*Colon*<br>*Sigmoid colon*<br>*Abdominal, unidentified*			 <br>3 (8.1%)<br>1 (2.7%)<br>5 (13.5%)<br>1 (2.7%)<br>6 (16.2%)<br>1 (2.7%)<br>18 (48.7%)<br>1 (2.7 %)<br>1 (2.7%)
**APACHEII score at admission**<br>mean (range) ± SD			18.65 (5-52) ± 7.75
**SOFA score at admission**<br>mean (range) ± SD			6.4 (2-12) ± 2.83
**Days in Surgical Critical Unit**<br>mean (range) ± SD			18.33 (2-167) ± 28.47
**Days of mechanical ventilation**<br>mean (range) ± SD			12.75 (0-167) ± 31.31
**Mortality** N (%)			12 (34.3%)

SD, standard deviation; *ns*, no significant difference (p> 0.05); Chi-square (χ^2) test was used, except for age, where a one-way ANOVA test was used.

Supplementary Table 2. Demographic and clinical data of septic patients. Related to Figures 1 and 2.

	**Septic patients**	**Septic patients with profound NLRP3 deactivation**
**N**	35	11
**Age**		
Mean (range) ± SD	69.33 (43-91) ± 13.34	74.64 (53-90) ± 11.64
p value against septic group		*p*> 0.05^{ns}
**Gender**, N (%)		
Male	21 (60)	6 (54.5)
Female	14 (40)	5 (45.5)
p value against septic group		*p*> 0.05^{ns}
**Oncologic patients**		
N (%)	11 (31.4)	6 (54.5)
p value against septic group		*p*> 0.05^{ns}
**Organ dysfunction**, N (%)		
Renal	17 (48.6)	6 (54.5)
Respiratory	24 (68.6)	9 (81.8)
Cardiovascular	26 (74.3)	10 (90.9)
Hepatic	16 (45.7)	4 (36.4)
Hematological	5 (14.3)	0 (0)
p value against septic group		*p*> 0.05^{ns}
**Infection acquired**, N (%)		
Nosocomial	19 (54.3)	7 (63.6)
Community	16 (45.7)	4 (36.4)
p value against septic group		*p*> 0.05^{ns}
**Mortality**		
N (%)	12 (34.3)	7 (63.6)
**Mortality cause**, N (%)		
Initial multi-organic failure*	6 (50)	3 (42.8)
Secondary complications**	6 (50)	4 (57.2)

SD, standard deviation; *ns*, no significant difference (p> 0.05) with Chi-square (χ^2) test.

*Mortality due to the initial organ dysfunction without clear recuperation after initial admission.

**Recuperation of initial organ dysfunction after admission, mortality due to acquisition of new infectious processes that could result in another multiple organ dysfunction.

Supplementary Table 3. Plasma cytokine levels (pg/ml) for septic individuals included in this study (ND: not determined). Related to Figure 1.

Individual	IL-1β	IL-2	IL-4	IL-6	IL-8	IL-12	IL-18	IFNγ	TNF-α	HMGB1
1	0	0	0	176.59	0	0	2193.11	0	0	29.32
2	115.88	0	0	238.33	22.05	0	772.39	0	139.25	ND
3	0	0	0	64.24	0	0	999.36	0	0	16.43
4	51.13	0	0	345.52	0	0	1065.07	0	600.94	26.22
5	0	0	0	158.36	0	0	418.82	0	0	25.78
6	0	0	0	88.66	0	0	661.32	0	0	20.89
7	0	0	0	53.73	0	0	1946.86	0	0	8.72
8	0	0	0	71.46	0	0	649.54	0	0	0
9	0	0	0	98.55	0	0	378.64	0	0	4.80
10	0	0	0	105.67	0	0	971.86	0	0	0
11	0	0	0	68.99	0	0	2288.46	0	96.20	13.34
12	81.43	0	0	311.52	0	0	271.86	0	0	1.33
13	0	0	0	228.74	0	0	336.14	0	0	5.13
14	0	0	0	113.33	0	0	723.46	0	0	9.80
15	0	0	0	80.08	0	0	439.00	0	0	7.90
16	796.60	0	0	763.26	0	0	516.87	0	0	6.38
17	0	0	0	754.84	34.05	0	1899.69	0	0	19.77
18	0	0	0	266.07	88.54	0	1318.44	0	0	16.21
19	0	713.82	0	2790.15	182.46	0	809.06	0	0	1.84
20	81.43	0	0	1416.87	0	0	466.87	0	0	8.01
21	0	0	0	691.51	0	0	663.75	0	0	5.26
22	0	0	0	ND	317.91	0	721.56	0	0	10.35
23	0	0	0	798.16	11.02	0	571.56	0	0	1.87
24	0	0	0	103.35	0	0	5584.06	0	0	10.31
25	488.95	ND	ND	137.62	121.92	ND	3254.36	ND	ND	8.16
26	1365.63	ND	ND	1583.34	142.22	ND	294.68	ND	ND	5.14
27	0	ND	ND	48.86	11.20	ND	1033.39	ND	ND	4.63
28	0	ND	ND	486.03	27.87	ND	549.52	ND	ND	3.84
29	0	ND	ND	507.71	166.07	ND	1786.61	ND	ND	3.21
30	732.58	ND	ND	910.04	97.84	ND	1454.36	ND	ND	5.24
31	201.55	ND	ND	109.60	6.24	ND	659.19	ND	ND	17.19
32	0	ND	ND	143.73	24.08	ND	1049.52	ND	ND	3.94
33	285.96	ND	ND	780.14	95.99	ND	1007.58	ND	ND	8.18
34	64.37	ND	ND	163.65	13.70	ND	723.71	ND	ND	8.80
35	49.73	ND	ND	492.43	36.76	ND	1278.55	ND	ND	8.40

Supplementary Table 4. Receiver operating characteristic (ROC) analysis for IL-1β, ASC-speck formation, HMBG1, IL-6, IL-8, TNF-α and ΔSOFA, comparing control vs septic individuals (Control) and septic individuals vs those septic individuals with a profound NLRP3 deactivation (Sepsis). Related to Figure 2.

	Median control	Median sepsis	ROC	AUC±SE	*p*	Cut-off	Sensitivity (%)	Specificity (%)
IL-1β	1121	264.3***	Control *vs* sepsis	0.83±0.06	0.00018	<856	78.2	76.2
			non-IC *vs* IC	0.98±0.02	<0.0001	<146	90.9	100
ASC-specks	31.55	8.6***	Control *vs* sepsis	0.95±0.03	<0.0001	<15.6	82.6	100
			non-IC *vs* IC	0.90±0.06	0.00111	<7.3	81.8	91.7
HMGB1	10.36	3.53^{ns}	Control *vs* sepsis	0.62±0.11	0.2482	<9.8	64.29	55
			non-IC *vs* IC	1	<0.0001	<3.5	100	100
IL-6	124	58.9*	Control *vs* sepsis	0.70±0.08	0.023	<70.9	60.9	73.7
			non-IC *vs* IC	1	<0.0001	<51.5	100	100
TNF-α	409.3	287.7^{ns}	Control *vs* sepsis	0.66±0.08	0.083	<253.5	65.2	73.7
			non-IC *vs* IC	1	<0.0001	<183.5	100	100
IL-8	872.2	1488^{ns}	Control *vs* sepsis	0.53±0.10	0.7918	>998.2	61.1	70.6
			non-IC *vs* IC	0.89±0.07	0.00576	<1243	85.7	81.8
ΔSOFA	ND	2	non-IC *vs* IC	0.92±0.07	0.00459	<1.35	87.5	87.5

AUC, area under the curve; SE, standard error; Control: ROC from control individuals *vs* septic patients; Sepsis: ROC from septic patients with a profound NLRP3 deactivation *vs* the rest of septic patients; ND, not determined; Mann-Whitney test to compare control *vs* sepsis: *p<0.05; ***p<0.0001; *ns*, no significant difference (p> 0.05). Control group included healthy donors and abdominal surgery controls (*n*= 27), and sepsis group included *n*= 23.

The median and cut-off values for IL-1β are expressed in pg/ml normalized to $5x10^4$ monocytes, for HMBG1 the value is ng/ml normalized to $5x10^4$ monocytes, for ASC-specks the value is the percentage of monocytes with intracellular ASC speck formation and for ΔSOFA the value is the variation of SOFA score between day 1 and 5 (SOFA day 1 – SOFA day 5).

Supplementary Table 5. Peritoneal microbial isolation at the moment of the initial surgery in septic patients. Related to Figure 2.

	**Septic patients**	**Septic patients with profound NLRP3 deactivation**
**Peritoneal isolation**, N (%)		
**Gram-negative bacteria**		
Escherichia coli	17 (48.6)	5 (41. 7)
Prevotella sp.	5 (14.3)	1 (8. 3)
Klebsiella sp.	5 (14.3)	3 (25)
Bacteroides sp.	5 (14.4)	1 (8.3)
Pseudomonas aeruginosa	2 (5.7)	1 (8.3)
Enterobacter sp.	2 (5.7)	0
Citrobacter sp.	2 (5.7)	0
Proteus mirabilis	1 (2.8)	0
**Gram-positive bacteria**		
Clostridium perfringens	1 (2.8)	0
Streptococcus sp.	8 (22.8)	3 (25)
Enterococcus sp.	8 (22.8)	1 (8.3)
Staphylococcus sp.	1 (2.8)	1 (8.3)
**Fungi**		
Candida sp.	4 (11.4)	1 (8.3)
**Negative**	3 (8.6)	0
p value against septic group		$p > 0.05^{ns}$

ns, no significant difference (p> 0.05) with Chi-square (χ^2) test.

SUPPLEMENTARY FIGURES

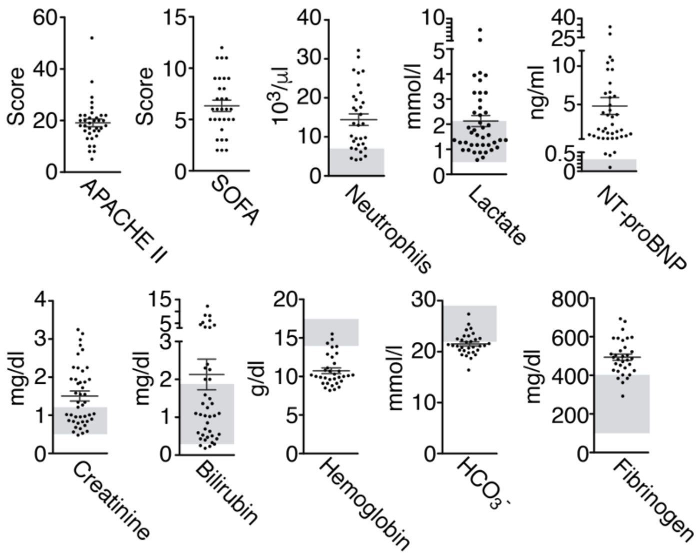

Supplementary Figure 1. Clinical parameters of septic patients within the first 24 h of admission to the surgical critical unit (day 1). SOFA and APACHEII clinical score from septic patients enrolled in our study. Number of circulating neutrophils, concentration of lactate, NT-proBNP, creatinine, bilirubin, haemoglobin, HCO_3^- and fibrinogen in peripheral blood samples. Grey shadow on graphs indicates the normal range in healthy population for each parameter analysed. Each dot represents an individual patient. Related to Figure 1.

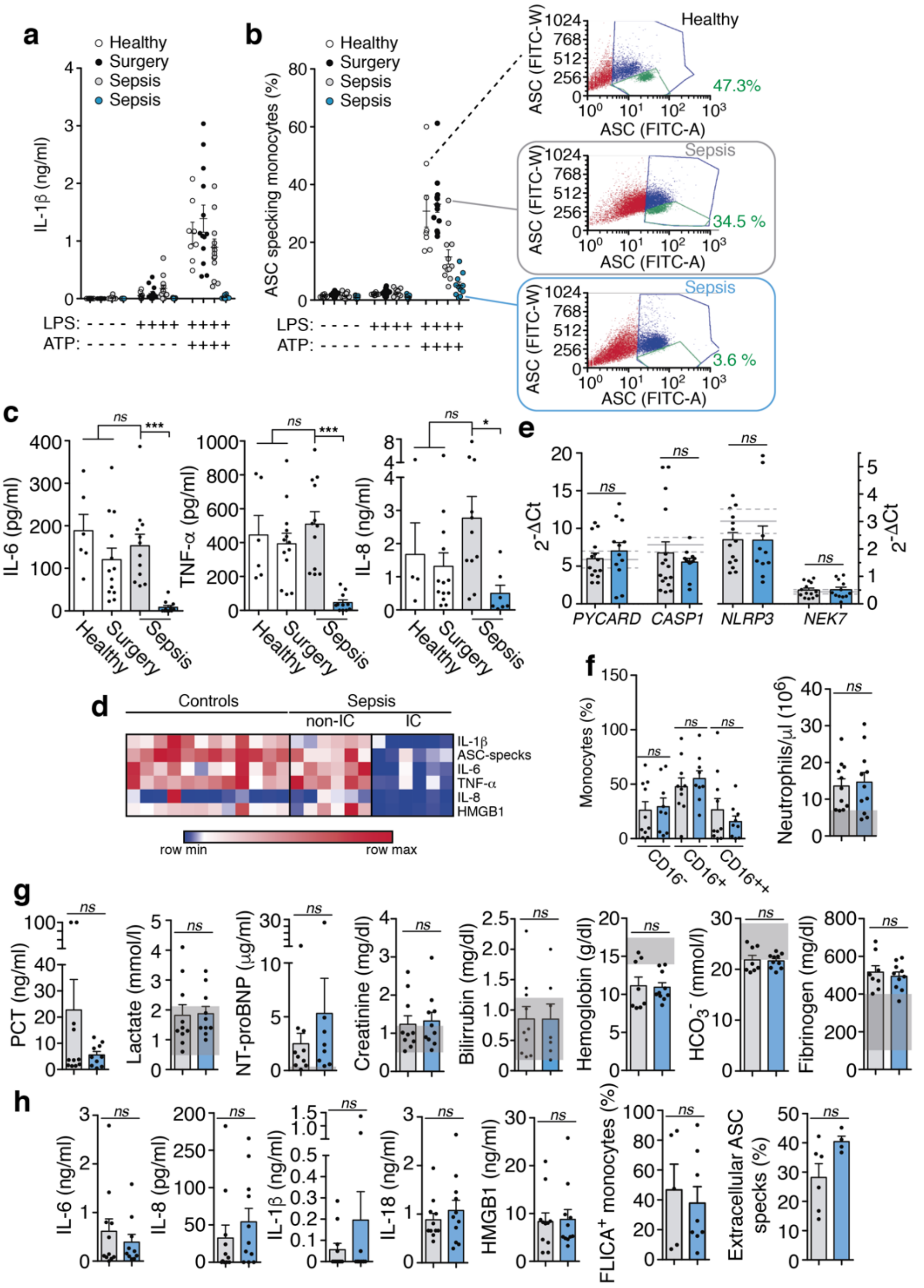

Supplementary Figure 2. Inflammatory and biochemical determinations in septic patients. (**a**) ELISA for IL-1β in PBMCs supernatants from septic patients at day 1 and control groups after NLRP3 inflammasome activation by LPS (1 μg/ml, 2h) and ATP (3 mM, 30 min) treatment. Blue represents septic patients with a profound deactivation of the NLRP3 inflammasome. (**b**) Percentage of monocytes with intracellular ASC specks from control groups and septic patients as in A (left), and representative dot-plots of ASC staining (FITC channel) wide (FITC-W) and area (FITC-A) (right) where green gate indicates monocytes with ASC specks; grey box represents a non-immunocompromised patient; blue box represents an immunocompromised patient. (**c**) Multiplexing for IL-6, TNF-α and IL-8 cytokines in PBMC supernatants from septic patients stratified as in main figure 2b and control groups after LPS (1 μg/ml, 2h) treatment. (**d**) Relative values of minimum and maximum concentrations of

cytokines and percentage of ASC specking monocytes; IC: immunocompromised septic patients. (**e**) Expression of *PYCARD* (ASC*)*, *CASP1*, *NLRP3* and *NEK7* genes analysed by quantitative PCR from septic patients' PBMCs at day 1; blue represents septic patients with a profound deactivation of the NLRP3 inflammasome; the average expression for surgery control group is represented by a continuous grey line for each gene; the s.e.m. for the surgery control group is represented by a dotted grey line for each gene. (**f**) Percentage of CD16 populations on $CD14^+$ monocytes (left) and number of circulating neutrophils (right) from septic patients at day 1; blue represents septic patients with a profound deactivation of the NLRP3 inflammasome. (**g**) Different clinical markers in the blood from septic patients at day 1; blue represents septic patients with a profound deactivation of the NLRP3 inflammasome; grey shadow on graphs indicates the normal range for each parameter analysed. (**h**) Cytokines measured from the plasma (IL-6, IL-8, IL-1β, IL-18 and HMGB1), percentage of monocytes with active caspase-1 ($FLICA^+$ monocytes) and presence of circulating ASC specks on plasma from septic patients at day 1; blue represents septic patients with a profound deactivation of the NLRP3 inflammasome. Each dot represents an individual patient; *ns*, no significant difference ($p > 0.05$); Mann-Whitney test for e-h. Related to Figure 2.

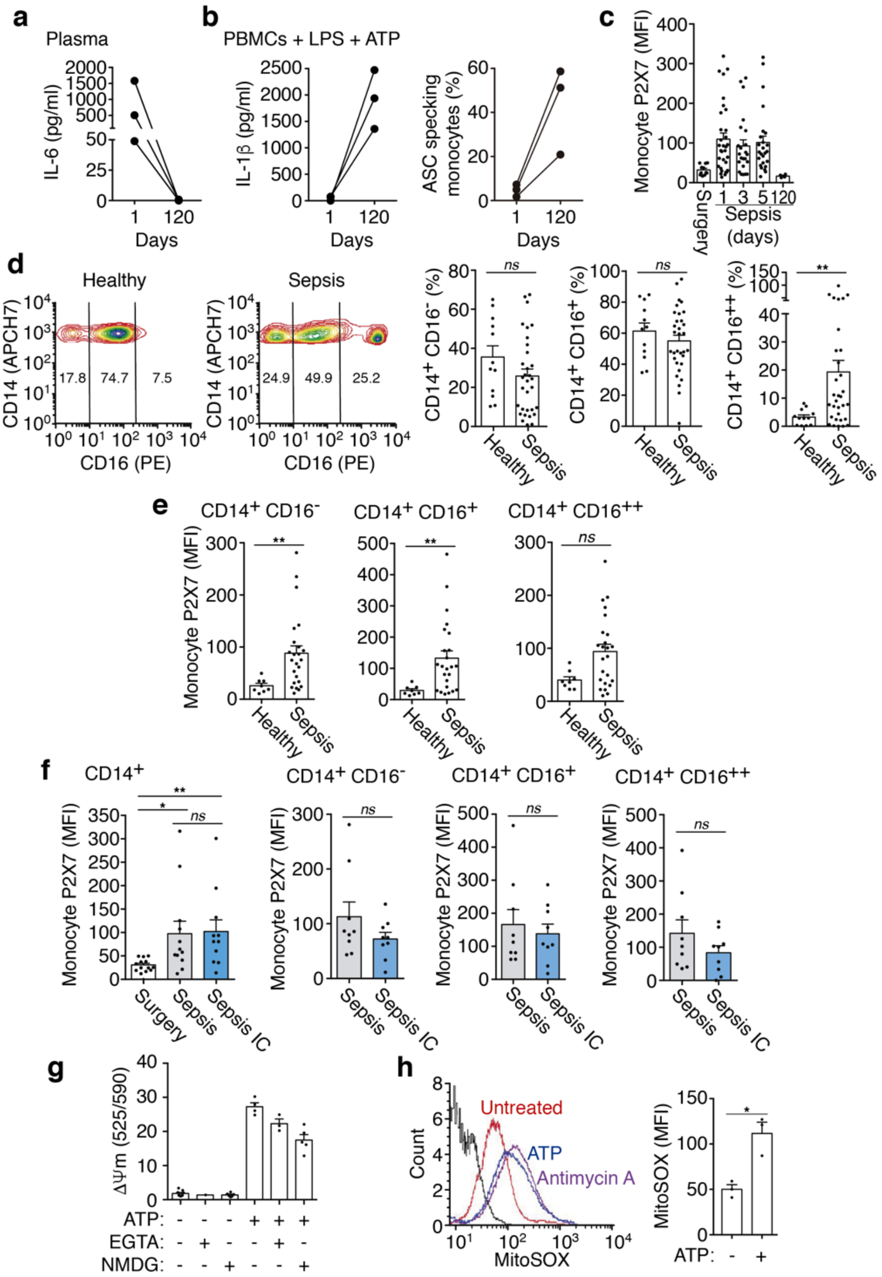

Supplementary Figure 3. NLRP3 inflammasome and P2X7 receptor in sepsis. (**a**) IL-6 concentration in plasma of septic patients at day 1 during sepsis and at day 120 after sepsis recovery. (**b**) ELISA for IL-1β in PBMC supernatants (left) and percentage of monocytes with intracellular ASC specks (right) after NLRP3 inflammasome activation by LPS (1 μg/ml, 2h) and ATP (3 mM, 30 min) treatment from the same patient group as in **a**. (**c**) Quantification of P2X7 receptor mean fluorescence intensity (MFI) in control and septic patients on different days. (**d**) Representative plot for $CD14^{+}CD16^{-/+/++}$ monocytes in a control healthy donor and in a septic patient at day 1 (top); and percentage of $CD14^{+}CD16^{-/+/++}$ monocyte populations from healthy individual and septic patients at day 1 (bottom); Numbers in top plots represent the percentage for the different CD16 populations. (**e**) Quantification of P2X7 receptor mean fluorescence intensity (MFI) in the different $CD16^{+}$ monocyte populations from patient groups as in **a**. (**f**) Quantification of P2X7 receptor mean fluorescence intensity (MFI) in control and septic patients

at day 1 in different monocyte subsets; blue represents septic patients with a profound deactivation of the NLRP3 inflammasome. (**g**) Mitochondrial membrane depolarization from wild-type BMDMs treated for 30 min with ATP (3 mM) in a Ca^{2+} free buffer (EGTA) or Na^{+} free buffer (NMDG). (**h**) Reactive oxygen species detection by MitoSOX staining in THP-1 cells untreated (red trace), ATP-treated (3 mM, 30 min, blue trace), or antimycin A-treated (5 μM, 30 min, purple trace); unstained THP-1 cells are shown as a black trace; each dot on the right histogram represents MitoSOX MFI from a single independent experiment. Each dot represents an individual patient (a-f) or a single independent experiment (g,h). $^{*}p < 0.05$; $^{**}p < 0.01$; *ns*, no significant difference ($p > 0.05$); Mann-Whitney test was used for d,f; *t*-test for h. Related to Figures 3, 4, and 5.

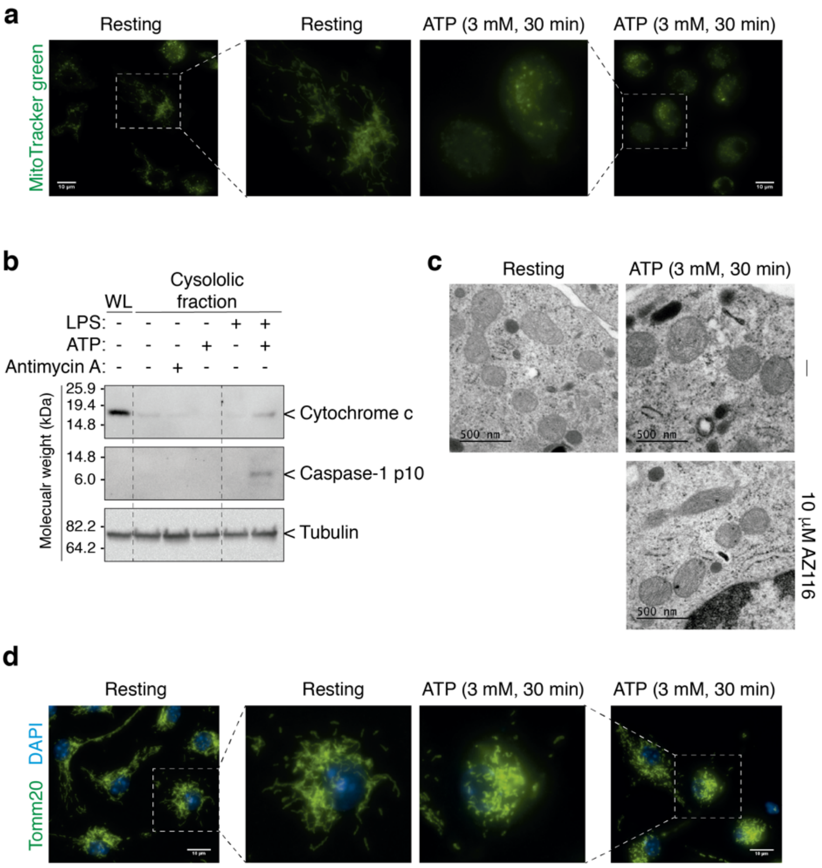

Supplementary Figure 4. Stimulation of P2X7 receptor does not induce mitochondrial damage. (**a**) Mitochondrial staining with Mitotracker-green of BMDM untreated (resting) or treated for 30 min with ATP (3 mM). (**b**) Immunoblot for cytochrome c, caspase-1 (p10) and tubulin in whole cell lysates (WL) or organelle-free cytosolic fraction from BMDMs primed or not for 4 h with LPS (10ng/ml) and then stimulated or not for 30 min with antymicin A (10 μM) or ATP (3 mM) as indicated. (**c**) Transmission electron microscopy from isolated human monocytes unstimulated (resting) or stimulated for 30 min with ATP (3 mM) in the presence or absence of the P2X7 receptor antagonist AZ11645373 (AZ116, 10 μM, 15 min before ATP and during ATP stimulation). (**d**) Tomm20 immunostaining (green) and nuclei (DAPI, blue) of BMDM untreated (resting) or treated for 30 min with ATP (3 mM). Related to Figure 5.

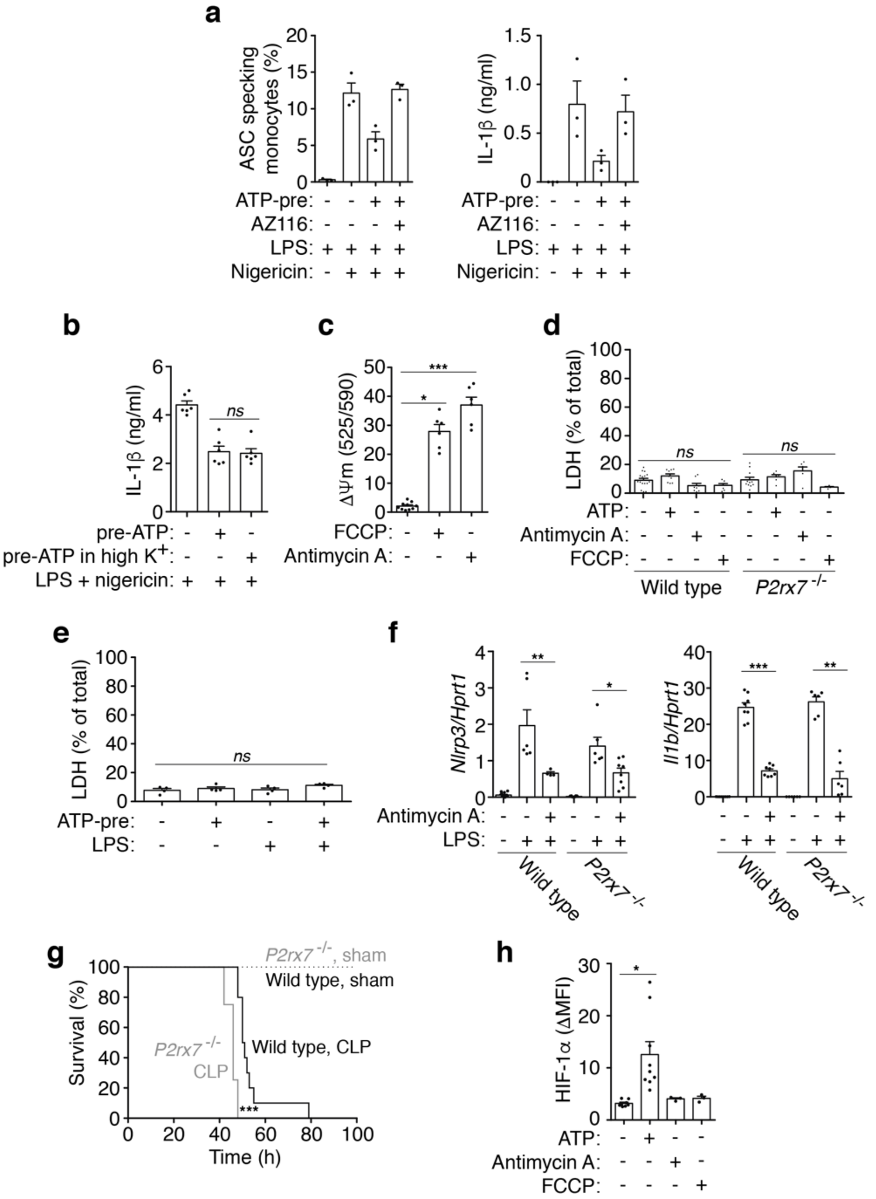

Supplementary Figure 5. Dysfunctional mitochondria do not induce cell death. (**a**) Percentage of monocytes with ASC-specks (left) and release of IL-1β from PBMCs (right) isolated from healthy donor blood samples treated with ATP (3 mM, 30 min; ATP-pre) in the presence or absence of the P2X7 receptor antagonist AZ11645373 (AZ116, 10 μM), then washed, primed with LPS (1 μg/ml, 2h) and then treated for 20 min with nigericin (10 μM) as indicated. (**b**) IL-1β release from BMDMs treated with ATP (3 mM, 30 min; ATP-pre) in normal buffer or high K^+ buffer (100 mM KCl) as indicated, then washed and primed with LPS (1 μg/ml, 4h) and then treated for 30 min with nigericin (10 μM) as indicated. (**c**) Mitochondrial depolarization on untreated, antimycin A-treated (5 μM, 30 min) or FCCP-treated (1 μM, 30 min) BMDMs. (**d**) Lactate dehydrogenase (LDH) detected in wild type or *P2rx7*$^{-/-}$ mouse BMDM supernatants treated for 30 min with ATP (3 mM), antimycin A (5 μM) or FCCP-treated (1 μM). (**e**) LDH detected in PBMC supernatants isolated from healthy donor blood samples treated with ATP (3 mM, 30 min; ATP-pre), then washed and primed with or without LPS (1 μg/ml, 4h). (**f**) Expression of *Nrlp3*, *Il1b* and *Il6* genes analysed by quantitative PCR from wild type or *P2rx7*$^{-}$

$^{-/-}$ BMDMs treated with antimycin A (5 μM, 30 min), then washed and primed with or without LPS (100 ng/ml, 4h). (**g**) Kaplan-Meier representation of CLP operated wild type (*n*= 10, black line) or *P2rx7*$^{-/-}$ (*n*= 3, grey line), or sham operated wild type (*n*= 4, black dotted line) or *P2rx7*$^{-/-}$ (*n*= 3, grey dotted line). (**h**) Quantification of HIF-1α mean fluorescence intensity increase (ΔMFI) in monocytes isolated from healthy donor blood samples and treated or not for 30 min with ATP (1 mM), antimycin A (5 μM) or FCCP (1 μM). Each dot represents a single independent experiment, except for panel e where each dot represent an individual healthy donor; *p< 0.05; **p< 0.01; ***p< 0.001; *ns*, no significant difference (p> 0.05); Mann-Whitney test for f; Kruskal-Wallis test for b-e,h; Long-rank test for g. Related to Figure 6 and 7.

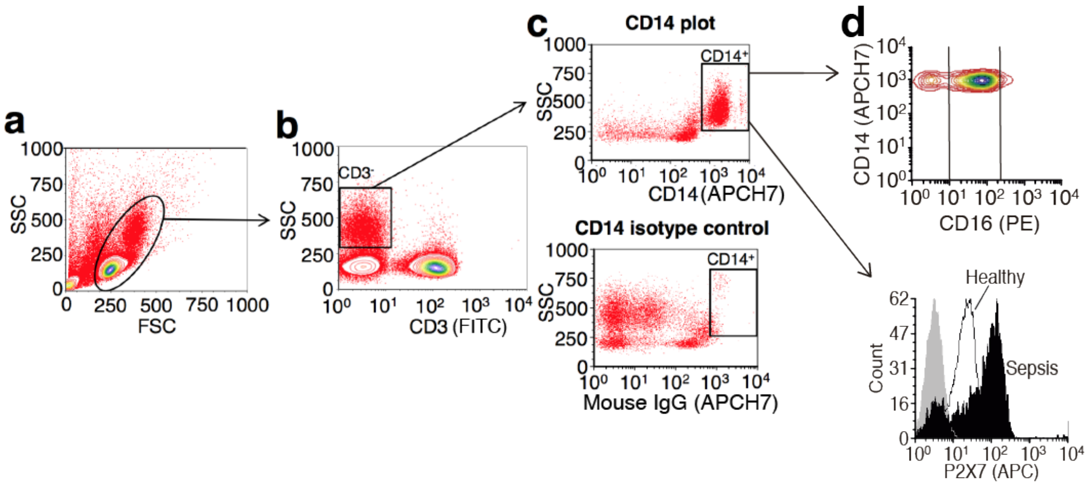

Supplementary Figure 6. Gating strategy used in flow cytometry experiments to detect $CD16^+$ monocytes and P2X7 receptor. (**a**) Representative dot-plot for PBMCs gating (the oval represents the viable initial PBMCs population gated) from singlets. (**b**) Representative SSC-CD3 dot-plot for PBMCs gated in **a**; $CD3^-$ cells with SSC^{high} were gated. (**c**) Representative SSC *vs* CD14 dot-plot for cells gated in **b**; $CD14^+$ cells were gated; top dot-plot is a stained sample, whereas bottom dot-plot represent a sample stained with antibody control isotype. (**d**) Representative CD14-CD16 dot-plot for cells gated in **c** as employed in Supplementary Figure 3 (top) or P2X7 histogram for cells gated in **c** as employed in Figure 4a,b,d-f (bottom).

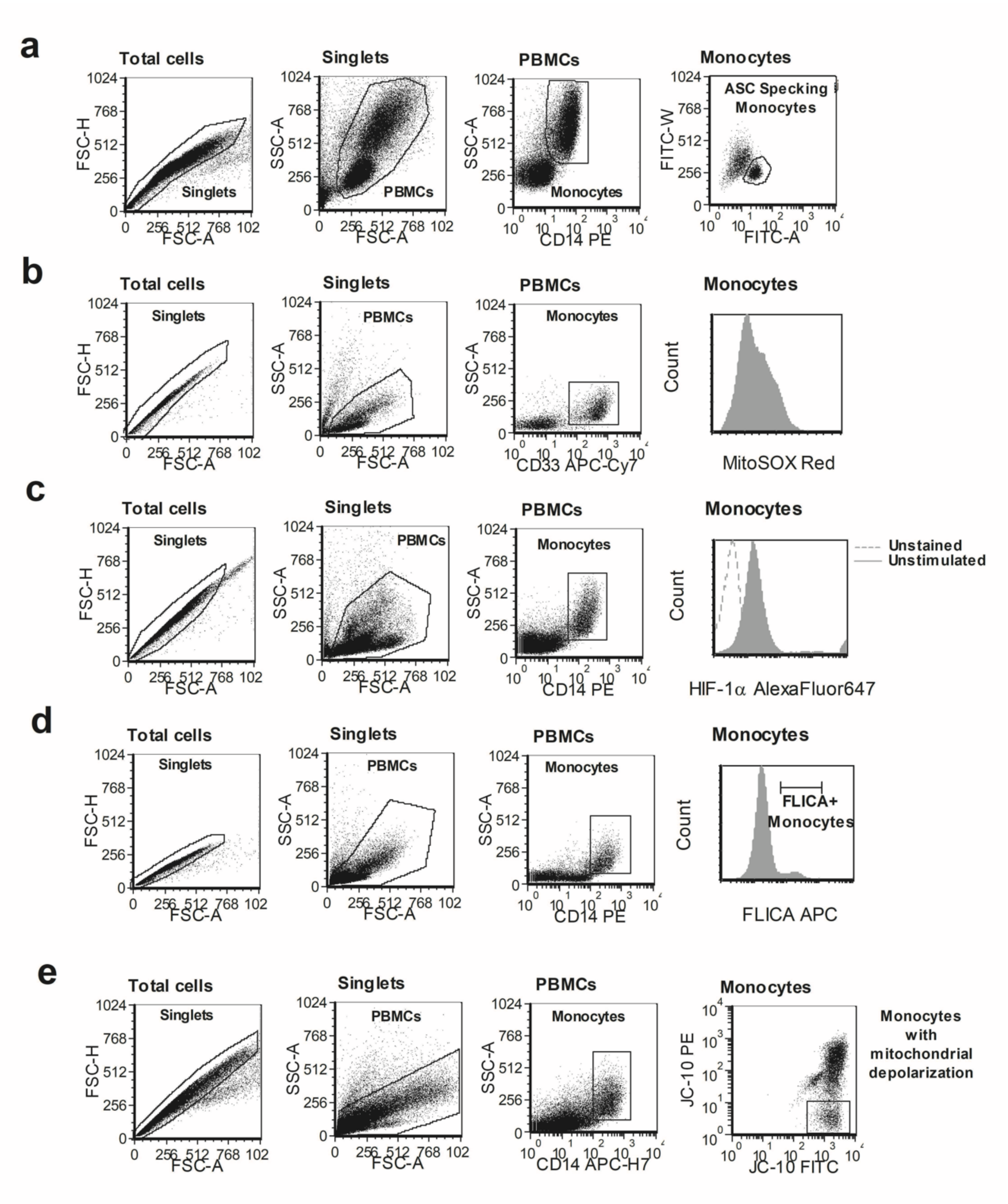

Supplementary Figure 7. Gating strategy used in flow cytometry experiments to detect intracellular ASC-specks, mitochondrial reactive oxygen production, HIF-1α, active caspase-1 and mitochondrial membrane potential. (**a**) Gating strategy used to quantify intracellular ASC specks in human monocytes as presented in Figures 1d, 2b, 3b and 6a. (**b**) Gating strategy used to determine mitochondrial reactive oxygen production by MitoSOX staining in human monocytes as presented in Figures 5f-g. (**c**) Gating strategy used to detect HIF-1a in human monocytes as presented in Figure 7e. (**d**) Gating strategy used to quantify caspase-1 activation by FLICA staining in human monocytes as presented in Figure 1d. (**e**) Gating strategy used to quantify mitochondrial depolarization by JC-10 staining in human monocytes as presented in Figure 5d-e.

Supplementary References

1. Single, B., Leist, M. & Nicotera, P. Differential effects of bcl-2 on cell death triggered under ATP-depleting conditions. Exp. Cell Res. 262, 8–16 (2001).
2. de Torre-Minguela, C., Barberà-Cremades, M., Gómez, A. I., Martín-Sánchez, F. & Pelegrín, P. Macrophage activation and polarization modify P2X7 receptor secretome influencing the inflammatory process. Sci. Rep. 6, 22586 (2016).
