# Supplementary material for: P2X7 receptor induces mitochondrial failure in monocytes and compromises NLRP3 inflammasome activation during sepsis
Source: Nat Commun. 2019 Jun 20;10:2711. doi: 10.1038/s41467-019-10626-x (PMC6586640; doi:10.1038/s41467-019-10626-x)
Supplement: Supplementary file 2 — Reporting Summary [file 41467_2019_10626_MOESM2_ESM.pdf]

nature research

Corresponding author(s): Pablo Pelegrín

Reporting Summary

Nature Research wishes to improve the reproducibility of the work that we publish. This form provides structure for consistency and transparency in reporting. For further information on Nature Research policies, see Authors & Referees and the Editorial Policy Checklist.

Statistical parameters

When statistical analyses are reported, confirm that the following items are present in the relevant location (e.g. figure legend, table legend, main text, or Methods section).

n/a	Confirmed	
☐	☒	The exact sample size (n) for each experimental group/condition, given as a discrete number and unit of measurement
☐	☒	An indication of whether measurements were taken from distinct samples or whether the same sample was measured repeatedly
☐	☒	The statistical test(s) used AND whether they are one- or two-sided<br>*Only common tests should be described solely by name; describe more complex techniques in the Methods section.*
☐	☒	A description of all covariates tested
☐	☒	A description of any assumptions or corrections, such as tests of normality and adjustment for multiple comparisons
☐	☒	A full description of the statistics including central tendency (e.g. means) or other basic estimates (e.g. regression coefficient) AND variation (e.g. standard deviation) or associated estimates of uncertainty (e.g. confidence intervals)
☐	☒	For null hypothesis testing, the test statistic (e.g. F, t, r) with confidence intervals, effect sizes, degrees of freedom and P value noted<br>*Give P values as exact values whenever suitable.*
☒	☐	For Bayesian analysis, information on the choice of priors and Markov chain Monte Carlo settings
☒	☐	For hierarchical and complex designs, identification of the appropriate level for tests and full reporting of outcomes
☐	☒	Estimates of effect sizes (e.g. Cohen's d, Pearson's r), indicating how they were calculated
☐	☒	Clearly defined error bars<br>*State explicitly what error bars represent (e.g. SD, SE, CI)*

Our web collection on statistics for biologists may be useful.

Software and code

Policy information about availability of computer code

Data collection	Gene5 & Gene 5 Secure (Biotek Instruments, USA), Image Lab 5.2.1 (Biorad, USA), iQ5 Optical System software v2.0 (Biorad, USA), FACS diva software (BD, Bioscience), Imaging Software NIS-Elements v4.3 (Nikon, Japan)
Data analysis	Prism (GraphPad Inc., v6 and v7), FCS Express 4 Flow Research (De novo software), ImageJ (US National Institutes of Health, Bethesda, MD USA), Gene5 & Gene 5 Secure (Biotek Instruments, USA), Morpheus (Broadinstitute).

For manuscripts utilizing custom algorithms or software that are central to the research but not yet described in published literature, software must be made available to editors/reviewers upon request. We strongly encourage code deposition in a community repository (e.g. GitHub). See the Nature Research guidelines for submitting code & software for further information.

Data

Policy information about availability of data

All manuscripts must include a data availability statement. This statement should provide the following information, where applicable:

- Accession codes, unique identifiers, or web links for publicly available datasets
- A list of figures that have associated raw data
- A description of any restrictions on data availability

Associated data for figures 1 to 7 is presented in the Source Data file. Other data are available from the corresponding author upon reasonable request.

Field-specific reporting

Please select the best fit for your research. If you are not sure, read the appropriate sections before making your selection.

☒ Life sciences ☐ Behavioural & social sciences ☐ Ecological, evolutionary & environmental sciences

For a reference copy of the document with all sections, see nature.com/authors/policies/ReportingSummary-flat.pdf

Life sciences study design

All studies must disclose on these points even when the disclosure is negative.

Sample size	The exact sample size is indicated for each particular panel in Source Data file. Sample size for septic patients was estimated before study begin by StatMate software (GraphPad Inc) to be >25 for a power >95% and significance of 0.025. Sample size for septic patients is n= 35. Sample size for in vitro assays and in vivo model were not estimated.
Data exclusions	Outliers from data sets were identified by the ROUT method with Q=1% using Prism v6 software (GraphPad Inc).
Replication	In vitro experiments were repeated at least two times. In the case of human samples, due to the limitation of the samples and the cell numbers, the experiment from each patient or donor was done from one to three technical replicates for each patient. Animal models were replicated at least two times.
Randomization	Samples for healthy, surgery controls and septic patients were randomized, as we were not planning the recollection of samples. Randomization was not applied for in vitro assays or in vivo models.
Blinding	Researchers were blind on the type of septic sample analyzed (immunocompromised or not), as this result was found once primary data was collected. They were not blinding respect the type of sample used (healthy, surgery or sepsis). Researchers were not blind for animal experiments. Not applied for in vitro assays.

Reporting for specific materials, systems and methods

Materials & experimental systems

n/a	Involved in the study	
☐	☒	Unique biological materials
☐	☒	Antibodies
☐	☒	Eukaryotic cell lines
☒	☐	Palaeontology
☐	☒	Animals and other organisms
☐	☒	Human research participants

Methods

n/a	Involved in the study	
☒	☐	ChIP-seq
☐	☒	Flow cytometry
☒	☐	MRI-based neuroimaging

Unique biological materials

Policy information about availability of materials

Obtaining unique materials	Unique materials were obtained from human individuals enrolled in this study. Excess of human plasma samples not used in this study are stored in the the Biobanco en Red de la Región de Murcia (PT13/0010/0018), integrated in the Spanish National Biobanks Network (B.000859) and are at disposition to other researchers upon reasonable request to the Biobank.

Antibodies

Antibodies used

Details of all antibodies used are described in the methods section and the on-line supplemental material and also listed here:

Antibody name: anti-ASC (N-15)-R rabbit polyclonal
Supplier name: Santa Cruz Biotechnology
Catalogue number: sc-22514-R
Lot number: #E1613
Dilution used: 1:1000

Antibody name: Alexa fluor 488 donkey anti-rabbit IgG (H+L)
Suplier name: Life Technologies
Catalogue number: A21206
Lot number: 1796375
Dilution used: 1:1000

Antibody name: PE-conjugated anti-human CD14 mouse monoclonal
Supplier name: TONBO Biosciences
Catalogue number: 50-0149-T025
Clone name: 61D3
Lot number: C0149081517503
Dilution used: 5ul /1.000.000 cells

Antibody name: APC-H7-conjugated anti-human CD14 mouse monoclonal
Supplier name: BD Pharmigen
Catalogue number: 560180
Clone name: MϕP9
Lot number: 5261916
Dilution used: 5ul /1.000.000 cells

Antibody name: FITC conjugated anti-human CD3 mouse monoclonal
Supplier name: Biolegend
Catalogue number: 35-0039-T100
Clone name: Hit3a
Lot number: C0039051215353
Dilution used: 5ul /1.000.000 cells

Antibody name: APC-Vio770-conjugated anti-human CD33 mouse monoclonal
Supplier name: Miltenyi Biotech
Catalogue number: 130-111-139
Clone name: REA775
Lot number: 5171218284
Dilution used: 5ul /1.000.000 cells

Antibody name: PE-Cy7-conjugated anti-human CD16 mouse monoclonal
Supplier name: BD pharmigen
Catalogue number: 557744
Clone name: 3G8
Lot number: 5357540
Dilution used: 5ul /1.000.000 cells

Antibody name: Alexa fluor 647-conjugated anti-human HIF-1alpha mouse monoclonal
Supplier name: BD pharmigen
Catalogue number: 565924
Clone name: 54/HIF-1alpha
Lot number: 7153517
Dilution used: 10ul /500.000 cells

Antibody name: anti-cytochrome c mouse monoclonal (7H8.2C12)
Suplier name: Abcam
Catalogue number: ab13575
Lot number: GR196392-43
Dilution used: 1:1000

Antibody name: anti-alpha tubulin
Suplier name: Abcam
Catalogue number: ab4074
Lot number: GR290489-2
Dilution used: 1:5000

Antibody name: anti-caspase-1 p10 (M-20)
Suplier name: Santa Cruz
Catalogue number: sc-514

Lot number: #LO215
Dilution used: 1:1000

Antibody name: Anti-TOMM20 [EPR15581-54]
Suplier name: Abcam
Catalogue number: ab186735
Lot number: GR322815-3
Dilution used: 1:250

Antibody name: ECL Anti-mouse IgG, horseradish peroxidase linked whole antibody
Suplier name: GE Healthcare
Catalogue number: NA931V
Lot number: 9557666
Dilution used: 1:5000

Antibody name: ECL Anti-rabbit IgG, horseradish peroxidase linked F(ab´)2 (from donkey)
Suplier name: GE Healthcare
Catalogue number: NA9340V
Lot number: 9784574
Dilution used: 1:5000

Antibody name: Alexa Fluor 647 F(ab´) 2 fragment goat anti-rabbit IgG (H+L)
Suplier name: Life Technologies
Catalogue number: A21246
Lot number: 1345057
Dilution used: 1:2000

Antibody name: anti-Asc, pAb (AL177)
Suplier name: Adipogen
Catalogue number: AG-25B-0006-C100
Lot number: A25281309
Dilution used: 1:1000

Validation

Antibody name: anti-P2X7 antibody (L4 clone)
Specificity: human
Application: Flow Cyt (Blood 92, 3521-3528, 1998).

Antibody name: anti-P2X7 nanobody (13A7 clone)
Specificity: mouse
Application: Blocking (Sci. Transl. Med. 8, 366ra162, 2016).

Antibody name: anti-P2X7 nanobody (14D5 clone)
Specificity: mouse
Application: Potentiation (Sci. Transl. Med. 8, 366ra162, 2016).

Antibody name: anti-ASC (N-15)-R rabbit polyclonal
Specificity: mouse, rat and human
Application: Flow Cyt (J. Immunol. 194, 455–462, 2014).

Antibody name: Alexa fluor 488 donkey anti-rabbit IgG (H+L)
Specificity: Rabbit
Application: IF, Flow cyt (Arth. rheumatol. 68(12):3035-3041, 2016)

Antibody name: PE-conjugated anti-human CD14 mouse monoclonal
Specificity: human
Application: Flow Cyt (J. Immunol. 194, 455–462, 2014).

Antibody name: APC-H7-conjugated anti-human CD14 mouse monoclonal
Specificity: human
Application: Flow Cyt (Science. 1990; 249(4975):1431-1433)

Antibody name: FITC conjugated anti-human CD3 mouse monoclonal
Specificity: human
Application: Flow cyt (2007. J. Immunol. 178:4786)

Antibody name: APC-Vio770-conjugated anti-human CD33 mouse monoclonal
Specificity: human
Application: Flow Cyt (J. Leukoc. Biol. 2006; 79(1): 46-58)

Antibody name: PE-Cy7-conjugated anti-human CD16 mouse monoclonal
Specificity: human
Application: Flow Cyt (Blood. 1996; 88(6):2358-2361)

Antibody: Alexa fluor 647-conjugated anti-human HIF-1alpha mouse monoclonal

Specificity: human
Application: Flow cyt (Mol Cell Biol. 2005; 25(13):5675-86)

Antibody name: anti-cytochrome c mouse monoclonal (7H8.2C12)
Specificity: Mouse
Application: WB (Cell Rep 7:2042-53 (2014))

Antibody name: anti-alpha tubulin
Specificity: Mouse
Application: WB (Nucleic Acids Res 42:3089-103 (2014))

Antibody name: anti-caspase-1 p10 (M-20)
Specificity: Mouse, human
Application: WB (Eur. J. Immunol. 40: 1545-1551.)

Antibody name: Anti-TOMM20 [EPR15581-54]
Specificity: Mouse,rat, human
Application: IF (Cancer Cell Int 18:117 (2018))

Antibody name: ECL Anti-mouse IgG, horseradish peroxidase linked whole antibody (from sheep)
Specificity: Mouse
Application: WB (Sci Rep. 2016 Mar 3;6:22586.)

Antibody name: ECL Anti-rabbit IgG, horseradish peroxidase linked F(ab´)2 (from donkey)
Specificity: Rabbit
Application: WB (Sci Rep. 2016 Mar 3;6:22586.)

Antibody name: Alexa Fluor 647 F(ab´) 2 fragment goat anti-rabbit IgG (H+L)
Specificity: Rabbit
Application: Flow Cyt (Oncotarget. 2016 Sep 27;7(39):63215-63225)

Antibody name: anti-Asc, pAb (AL177)
Specificity: Human, Mouse
Application: Flow Cyt (Nat Immunol. 2014 Aug;15(8):738-48.)

Eukaryotic cell lines

Policy information about cell lines

Cell line source(s)	THP-1 cells (TIB-202; American Type Culture Collection).
Authentication	THP-1 cell line used was no authenticated
Mycoplasma contamination	THP-1 cells were routinally tested for Mycoplasma contamination and were clean for Mycoplasma
Commonly misidentified lines (See ICLAC register)	No commonly misidentified cell lines were used.

Animals and other organisms

Policy information about studies involving animals; ARRIVE guidelines recommended for reporting animal research

Laboratory animals	We worked with bone marrow-derived macrophages obtained from laboratory mice (Mus musculus) wild-type C57BL/6J background, or from NOD.129P2(B6)-P2rx7tm1Gab (P2X7-/-), C57 BL/6 Nlrp3tm1Vmd (NLRP3-/-) or B6N.129S2-Casp1tm1Flv/J (Caspase1-/-). Males and females were used for bone marrow extraction at the age of 8-10 weeks. Males at the age of 8-10 weeks were used for the cecal puncture and ligation protocol.<br>The University of Murcia Animal Research Ethical Committee approved animal procedures (ref. 5/2014) and then the Health Animal Service, Fishing and Farming Council of Murcia (Servicio de Sanidad Animal, Dirección General de Ganadería y Pesca, Consejería de Agricultura y Agua Región de Murcia) approved animal procedures with ref. A1320140201.
Wild animals	none
Field-collected samples	none

Human research participants

Policy information about studies involving human research participants

Population characteristics	Healthy donors: n= 11<br>Abdominal surgery controls: n= 14<br>Septic patients: n= 35<br>Full demographics and clinical characteristics of the different populations is presented in Supplementary Table 1.

Recruitment

Informed consent was obtained from all individuals enrolled in the study following the principles set out in the WMA Declaration of Helsinki and samples were stored in the Biobanco en Red de la Región de Murcia (PT13/0010/0018) integrated in the Spanish National Biobanks Network (B.000859).
The Institutional Review Board of the Hospital Clínico Universitario Virgen de la Arrixaca approved the use of these blood samples.

Flow Cytometry

Plots

Confirm that:

☒ The axis labels state the marker and fluorochrome used (e.g. CD4-FITC).

☒ The axis scales are clearly visible. Include numbers along axes only for bottom left plot of group (a 'group' is an analysis of identical markers).

☒ All plots are contour plots with outliers or pseudocolor plots.

☒ A numerical value for number of cells or percentage (with statistics) is provided.

Methodology

Sample preparation

PBMCs from healthy blood donors or patients were isolated from whole peripheral blood using Histopaque-1077 (Sigma-Aldrich) and Cells were treated with ATP (Sigma-Aldrich), antimycin A (Sigma-Aldrich) or FCCP (Sigma-Aldrich) in the presence or absence of PDTC (Sigma-Aldrich) or echinomycin (Sigma-Aldrich) in E-total buffer (147 mM NaCl, 10 mM HEPES, 13 mM glucose, 2 mM CaCl2, 1 mM MgCl2, and 2 mM KCl) and then washed and stimulated with E. coli LPS O55:B5 in their respective complete media. In some experiments, PBMCs were activated with recombinant human IL-6, TNF-a or IFNg (PeproTech). After LPS treatment, cells were incubated with P2X7 modulating nanobodies, the specific P2X7 receptor antagonist AZ11645373 (Tocris), and then subsequent stimulated with ATP or nigericin (Sigma-Aldrich) in E-total buffer. Times and concentrations for the reagents used are specified in the figure legends. Intracellular ASC-speck formation was evaluated by the by Time of Flight Inflammasome Evaluation in CD14+ monocytes (anti-CD14PE clone61D3, Tonbo biosciencies) using a polyclonal unconjugated rabbit anti-ASC (N-15)-R antibody (SantaCruz Biotechnology) and a secondary monoclonal donkey anti-rabbit antibody Alexa Fluor-488 (Life Technologies). HIF-1a expresion was measured in CD33+ monocytes (anti-CD33 APC-vio770 clone REA775, Miltenyi biotech) using Alexa Fluor-647 mouse anti-human HIF-1a (BD Biosciences, Clone 54). Monocytes from PBMCs were determined by CD3-CD14+ selection (Supplementary Figure 6). P2X7 receptor surface expression was determined in CD16-/+/++ monocytes using the monoclonal anti-P2X7 L4 clone conjugated with APC. Active caspase-1 was measured in monocytes using the specific fluorescent probe FLICA-660 Caspase-1 Assay Kit (Immunochemistry Technologies) following manufacturer’s instructions. Production of ROS was measured in monocytes using the red mitochondrial superoxide indicator MitoSOX (Life Technologies) following manufacturer’s instructions. The detection of extracellular particles of ASC was performed on 0.5 ml of human plasma by flow cytometry using a polyclonal unconjugated rabbit anti-ASC (AL177) antibody (Adipogen) at 1:1000 dilution and a secondary Alexa Fluor 647 F(ab') fragment of goat anti-rabbit IgG (H+L) (Life Technologies) at 1:2000 dilution (Baroja-Mazo et al. Nat Immunol 2014).

Instrument

FACS Canto cytometer (BD Biosciences)

Software

FACS diva software (BD Biosciences) & FCS Express 4 Flow Research (De novo software)

Cell population abundance

Monocytes were the population of interest and accounted between 15-20% of PBMCs after Ficoll gradient isolation from whole blood.

Gating strategy

For P2X7 expression and CD14/CD16 plots, monocytes were gated using SSC vs FCS, SSC vs CD3- (FITC), and SSC vs CD14+ (APCH7), and CD14+ (APCH7) vs CD16 (PE), and then P2X7+ (APC).

For ASC specking monocytes, monocytes were gated from PBMCs using SSC vs CD14-PE dot plot, and then ASC specking monocytes were gated in a ASC-FITC-Width vs ASC-FITC-Area dot plot.

For HIF1a expression monocytes were gated from PBMCs using SSC vs CD14-PE dot plot, and then HIF-1a (APC) MFI was measured using a histogram plot.

For MitoSOX staining monocytes were gated from PBMCs using SSC vs CD33 APC-Cy7 dot plot, and then MitoSOX (PE) MFI was measured using a histogram plot.

For FLICA staining monocytes were gated using SSC vs FCS, SSC vs CD3- (FITC), and SSC vs CD14+ (APCH7) dot plots, and then FLICA (APC) positive monocytes were measured using a histogram plot.

For JC10 staining monocytes were gated using SSC vs FCS, and then SSC vs CD14+ (APCH7) dot plots.

☒ Tick this box to confirm that a figure exemplifying the gating strategy is provided in the Supplementary Information.
